# Current Landscape of Cancer Immunotherapy: Harnessing the Immune Arsenal to Overcome Immune Evasion

**DOI:** 10.3390/biology13050307

**Published:** 2024-04-28

**Authors:** Ankita Mitra, Anoop Kumar, Nitin P. Amdare, Rajiv Pathak

**Affiliations:** 1 Laura and Isaac Perlmutter Cancer Center, New York University Langone Medical Center, New York, NY 10016, USA; 2 Molecular Diagnostic Laboratory, National Institute of Biologicals, Noida 201309, Uttar Pradesh, India; 3 Department of Microbiology and Immunology, Albert Einstein College of Medicine, Bronx, New York, NY 10461, USA; 4 Department of Genetics, Albert Einstein College of Medicine, Bronx, New York, NY 10461, USA

**Keywords:** cancer immunotherapies, immune evasion, antibody-based therapy, adoptive cell transfer therapy, cancer vaccines, cytokine-based therapies, oncolytic viruses, metabolic reprogramming, hot and cold tumors, cancer immunotherapeutic resistance

## Abstract

**Simple Summary:**

Cancer immune evasion poses a significant challenge in developing effective cancer treatments. The interplay between the immune system and tumors influences cancer growth at various stages. While traditional treatments like surgery, radiation, chemotherapy, and targeted drugs remain fundamental, the emergence of immunotherapy has revolutionized cancer care. Harnessing the body’s immune system, immunotherapy significantly improves patient outcomes, prompting efforts to enhance treatment efficacy. This review explores recent advancements in understanding cancer immune evasion mechanisms and evaluates various immunotherapy approaches, including cancer vaccines, adoptive cell therapy, and antibody-based treatments. It covers a broad spectrum of treatments, from established ones to those still under investigation, offering important insights for developing new anticancer immunotherapies. By analyzing resistance mechanisms, exploring combination strategies, and employing personalized approaches, the review aims to illuminate pathways for more effective treatments. Overall, this review aims to summarize recent developments in cancer immunology and immunotherapy, detailing new insights into cancer immune evasion mechanisms for identifying novel treatments.

**Abstract:**

Cancer immune evasion represents a leading hallmark of cancer, posing a significant obstacle to the development of successful anticancer therapies. However, the landscape of cancer treatment has significantly evolved, transitioning into the era of immunotherapy from conventional methods such as surgical resection, radiotherapy, chemotherapy, and targeted drug therapy. Immunotherapy has emerged as a pivotal component in cancer treatment, harnessing the body’s immune system to combat cancer and offering improved prognostic outcomes for numerous patients. The remarkable success of immunotherapy has spurred significant efforts to enhance the clinical efficacy of existing agents and strategies. Several immunotherapeutic approaches have received approval for targeted cancer treatments, while others are currently in preclinical and clinical trials. This review explores recent progress in unraveling the mechanisms of cancer immune evasion and evaluates the clinical effectiveness of diverse immunotherapy strategies, including cancer vaccines, adoptive cell therapy, and antibody-based treatments. It encompasses both established treatments and those currently under investigation, providing a comprehensive overview of efforts to combat cancer through immunological approaches. Additionally, the article emphasizes the current developments, limitations, and challenges in cancer immunotherapy. Furthermore, by integrating analyses of cancer immunotherapy resistance mechanisms and exploring combination strategies and personalized approaches, it offers valuable insights crucial for the development of novel anticancer immunotherapeutic strategies.

## 1. Introduction

Cancer immune evasion presents a significant obstacle in the development of effective anticancer therapies. Despite advancements in understanding tumor immune escape mechanisms, efforts to counteract tumor evasion strategies have lagged behind. Several factors contribute to tumor persistence despite the presence of a functional immune system. Recent years have witnessed a shift towards focusing on the interplay between the immune system and cancer, leading to groundbreaking progress in cancer immunotherapy. This shift has been driven by a deeper understanding of the complex dynamics within the tumor microenvironment (TME), which comprises cancer cells, stromal cells, immune cells, and various soluble factors [1,2,3,4,5]. Within this milieu, tumor cells utilize diverse mechanisms to evade immune surveillance and establish an immunosuppressive environment, facilitating tumor growth and metastasis [6,7]. Among the key regulators of this dialogue are immune checkpoint pathways, which serve as critical modulators of immune responses and play pivotal roles in maintaining self-tolerance and preventing autoimmunity. Immune checkpoint pathways, including adaptive and innate checkpoints, represent fundamental mechanisms of immune regulation. Among these pathways, the programmed death-ligand 1 (PD-L1)/programmed cell death protein 1 (PD-1) axis and cytotoxic T-lymphocyte-associated protein 4 (CTLA-4) pathway have garnered significant attention due to their roles in immune evasion and tumor progression [8,9,10,11,12,13]. Moreover, emerging evidence suggests the involvement of additional checkpoint molecules, such as lymphocyte-activation gene 3 (LAG-3), T-cell immunoreceptor with Ig and ITIM domains (TIGIT), and V-domain Ig suppressor of T-cell activation (VISTA), in modulating immune responses within the TME [14,15,16,17,18].

The widespread commercial approval of genetically engineered T cells for a variety of blood cancers offers hope for patients with other types of cancer; cell-based immunotherapies (CBIs) are groundbreaking approaches in cancer treatment, harnessing the immune system to target malignancies [19,20,21,22]. These therapies involve modifying a patient’s immune cells ex vivo, such as T cells and natural killer (NK) cells, to enhance their ability to recognize and destroy cancerous cells. Notable examples include chimeric antigen receptor (CAR)-engineered T (CAR-T)-cell therapy and CAR-engineered NK (CAR-NK)-cell therapy, which have demonstrated remarkable efficacy against previously resistant cancers [19,20,21,23,24]. However, immune evasion by tumor cells presents a significant challenge to the effectiveness of CBIs [25]. Tumors evade or suppress the immune response by altering antigen expression, upregulating inhibitory molecules like PD-L1, and creating an immunosuppressive TME [26]. Overcoming these evasion tactics is crucial for optimizing CBI outcomes and achieving lasting anti-tumor responses. Crucially, tumor cells evade immune recognition by downregulating major histocompatibility complex (MHC) molecules and antigen processing machinery while also upregulating immune checkpoint ligands, hindering effective anti-tumor responses [27,28]. The past two decades have witnessed a transformative shift in medical paradigms towards personalized medicine, particularly evident in oncology with the advent of treatments targeting genomic alterations [29]. Concurrently, the breakthrough of checkpoint blockade immunotherapy has revolutionized cancer care by elucidating the intricate interplay between tumors and the host immune system. CD8^+^ cytotoxic T cells, pivotal in tumor shrinkage, recognize and eliminate cancer cells presenting tumor-specific antigens, albeit often encountering exhaustion due to inhibitory receptor ligands like PD-L1 or PD-L2 [8,9]. This immune activation paradoxically triggers the accumulation of CD4^+^ regulatory T cells (Tregs), dampening effector T-cell function [30,31]. Antibody-based checkpoint blockade immunotherapy enhances immune responses against tumors, with anti-CTLA-4 notably modulating CD4^+^ T-cell subsets to bolster effector T cells while restraining Treg activity [32,33,34]. Tumor neoantigens, arising from genetic alterations during tumorigenesis, have also emerged as promising targets [35,36]. These neoantigens, stemming from diverse genomic processes such as missense mutations, fusion transcripts, frameshifts, and stop losses, encode novel amino acid sequences [36,37,38]. Advances in next-generation sequencing (NGS) and bioinformatics have expedited neoantigen discovery, employing whole-exome sequencing and peptide translation alongside immunogenicity prediction algorithms for personalized neoantigen assessment, thus ushering in a new era of precision oncology [39,40].

While immune checkpoint inhibitors (ICIs) hold promises for long-term efficacy, their overall benefits remain limited due to low response rates in common tumor types like breast and prostate cancer and variations in response among different tumor lesions in the same patient. Immunotherapy faces significant challenges, with most patients either showing no response or developing resistance over time, whether through primary or acquired mechanisms [29,41]. Overcoming these challenges requires a deep understanding of the complex mechanisms of drug resistance in cancer immunotherapy and the adoption of effective combination therapy strategies. This review systematically explores the wide range of immune evasion mechanisms employed by tumor cells, followed by various immunotherapeutic strategies highlighting how these agents modify the immune response to combat cancer. The review addresses both established therapies and those currently under investigation, providing a comprehensive overview of ongoing efforts and highlighting the successful shift to immunotherapy. Furthermore, we discuss several challenges, such as spatial immune cell heterogeneity, alterations in the TME, antigen presentation, and diverse signaling pathways. Overall, this review offers valuable insights for developing novel therapeutic approaches to overcome immune evasion.

## 2. Mechanisms of Immune Suppression

Cancer progression involves distinct immune stages, including initial immune surveillance, followed by an equilibrium phase, and eventual immune suppression that promotes tumor growth [42,43]. When formulating a robust immunotherapeutic strategy, it is crucial to comprehend tumor–immune interactions. Tumors can be stratified based on an ImmuneScore [44]. The ImmuneScore, initially defined in colorectal cancer, quantitatively assesses the infiltration of CD3 and CD8 T cells in both the tumor center (CT) and the invasive margin (IM). This scoring system evaluates the immune status of the tumor based on the presence of Th1 and cytotoxic CD8 T cells, along with the expression of immune checkpoints and inflammatory and cytotoxic cytokines associated with T cells. Validated across cancers, this score predicts immunotherapy outcomes and correlates with prolonged survival [4,12,45,46]. Additionally, tumors are categorized as immune cold/silent, immune hot, and immune altered (suppressed/excluded) based on immune cell infiltration, providing a simplified framework for therapeutic approaches [5,44,47]. Immune hot tumors, with high ImmuneScore, exhibit extensive infiltration of effector immune cells within the tumor and activation of IFN-γ-induced signaling pathways, such as PD-L1. Additionally, they show increased expression of Foxp3, IDO, cytotoxic effector molecules, and enhanced antigen presentation, along with high tumor mutational burden (TMB) in some cases. In contrast, immune cold tumors, with low ImmuneScore, lack immune cell infiltration and generally display low immunogenicity and low TMB [5,44]. In immune-excluded tumors, tumor infiltration is confined mainly to the tumor stroma, excluding the core or parenchyma. This indicates the immune system’s potential to mount an effective response, which is, however, impeded by physical barriers imposed by the tumor to evade immune surveillance [48]. Conversely, in immune-altered suppressed tumors, there is minimal immune infiltration, signaling an immunosuppressive environment that hampers further immune cell infiltration and proliferation. Although all these systems define tumor immune status mostly based on the cytotoxic T-cell population, there are other components of the immune system to consider.

Immune surveillance within tumors involves both the innate and adaptive components of the immune system [49]. The primary challenge in anti-tumor immunity within the TME is the exhaustion and eventual dysfunction of immune cells, impacting their effector functions. This phenomenon is well described in the context of T cells, where the activation framework follows a three-signal system. Signal 1 involves the antigenic peptide bound to the MHC, signaling through the T-cell receptor. Signal 2 comprises co-stimulatory signals, while Signal 3 involves cytokines [50,51]. In contrast to co-stimulators, co-inhibitory receptors act as gatekeepers of T-cell activation, regulating the duration of activation and influencing cell differentiation and function. However, in the TME, this system of inhibitory signals is exploited to suppress the optimal functioning of immune cells. Although the immune status of tumors has traditionally been strongly associated with T-cell populations, TME incorporates several other factors, such as metabolic reprogramming, as a secondary method of immune suppression. This includes the recruitment and differentiation of immunosuppressive cells like myeloid-derived suppressor cells, regulatory T cells, and tumor-associated macrophages (TAMs). It is crucial to recognize that while certain tumors are conventionally categorized as “hot” or “cold”, the process of immune cell infiltration is inherently dynamic, both spatially and temporally. Improved biomarkers are needed to categorize patients based on tumor immune profiles, guiding the selection of immunotherapies and combinations tailored to the tumor’s immune milieu.

### 2.1. Immune Checkpoints and Cancer Immunotherapies

Co-inhibitory signaling operates through a variety of receptors commonly referred to as immune checkpoints. In healthy tissue, these checkpoints play a crucial role in maintaining tolerance and serve as brakes to regulate the activity and function of immune cells. However, within the TME, these checkpoints significantly contribute to T-cell exhaustion and dysfunction. Recently, Dutta et al. conducted a comprehensive review focusing on approved immune checkpoints and highlighted newer emerging inhibitors [13]. Here, we are providing an overview of both adaptive and innate immune checkpoints associated with the inhibition of immune cell function, highlighting recent advancements in the field.

#### 2.1.1. PD-1 and PD-L1

Programmed death protein-1 (PD-1/CD279) is an immune-inhibitory cell surface receptor and is expressed on various immune cells, including T cells, NK cells, B cells, dendritic cells (DCs), and monocytes. It interacts with its ligands, programmed death ligand-1 (PD-L1/B7-H1/CD274), or programmed death ligand-2 (PD-L2/B7-DC/CD273), which are expressed on macrophages, some activated T cells, and B cells, DCs, and some epithelial cells [10,52]. PD-1 signaling targets the TCR signaling pathway and the costimulatory CD28 pathways, with its signaling partner being SHP-2. Upon activation of the TCR signaling pathway, PD-1 is upregulated and initiates a signaling axis that leads to the inhibition of the PI3K/AKT signaling downstream of the TCR. This inactivation downregulates the expression of cell survival genes and promotes T-cell apoptosis while simultaneously inhibiting the secretion of various cytokines and effector molecules such as Granzymes, perforin, TNF, and IFN by the T cells [52,53]. PD-L1 expression on malignant cells is predominantly regulated by class I and II interferons through the JAK/STAT signaling axis, particularly IFN-γ. This leads to a phenomenon called “adaptive resistance” in tumors, wherein tumor cells adapt to evade immune surveillance by upregulating PD-L1 in response to IFN-γ secreted by tumor-infiltrating lymphocytes (TILs) [52,54]. PD-L1 expression serves as a measure of the tumor’s immunogenicity and can influence the outcome of PD-1 blockade-based therapies. However, in some cancers, upregulation of PD-L1 on cancer cells can occur independently of TIL presence. This independence can be attributed to factors such as loss of phosphatase and tensin homolog (PTEN), constitutive anaplastic lymphoma kinase (ALK) signaling, and EGFR mutations [55]. These factors may elucidate the failure of ICIs targeting PD-1/PD-L1 in certain cases.

#### 2.1.2. Cytotoxic T-Lymphocyte Antigen-4 (CTLA-4)

Unlike PD-1, which interacts with separate ligands for its inhibitory function, CTLA-4 binds to B7 family ligands used for costimulatory signaling, namely B7.1 (CD80) and B7.2 (CD86). CTLA-4, a homolog of the CD28 family with higher avidity for B7, is expressed on activated T cells and Foxp3^+^ regulatory T cells (Tregs), along with some expression on B cells, fibroblasts, and granulocytes [56]. During early T-cell priming in the lymph nodes, CTLA-4 prevents the formation of potentially autoreactive T cells [57]. However, its high avidity for CD80/CD86 allows it to compete with CD28, indirectly diminishing signaling through CD28, compromising the activation, proliferation, and cytokine secretion by the T cells [58]. With activation, CTLA-4 contained in the extracellular vesicles is trafficked to the synapse, stabilized by B7, and thus outcompetes and limits downstream signaling through CD28. Within the T cells, CTLA-4 interacts with the T-cell activation machinery such as PI3K, GRB2, filamin A, PKCθ, ZAP70, and phosphatases such as PTPN11 and PP2A, to initiate an inhibitory signal. Another potential mechanism of CTLA-4-mediated inhibition of T-cell activation and function involves the removal of costimulatory molecules on the antigen-presenting cell (APC) surfaces via transendocytosis, resulting in impaired T-cell costimulation—a cell-extrinsic function of CTLA-4 [11,59].

#### 2.1.3. Lymphocyte-Activation Gene 3 (LAG-3)

LAG-3, also known as CD223, is a coinhibitory receptor expressed on T cells upon activation, as well as on γδ T cells, B cells, and NK cells. It has been shown that LAG-3 is essential for the immunosuppressive function of Foxp3^+^ Tregs and IL-10-producing Foxp3- Type 1 regulatory Tregs (Tr1). The expression of LAG-3 is modulated by various factors, including persistent antigen exposure along with cytokines such as IL-2, IL-7, and IL-12 [60]. Initially, the structural homology between LAG-3 and CD4, with MHCII as the ligand for LAG-3, led to the hypothesis that LAG-3 functions by disrupting the MHCII:CD4 complex. However, the subsequent structural analysis revealed that its action involves suppressing T-cell responsiveness to stable MHC II:peptide complexes [61]. Other ligands, including fibrinogen-like protein 1 (FGL1), galectin-3 (Gal-3), and liver sinusoidal endothelial cell lectin (LSECtin), have been identified to bind with LAG-3, thereby suppressing CD8-mediated anti-tumor responses [62,63,64]. FGL1, expressed by tumors, has been associated with a poor prognosis in melanoma and NSCLC, and it has been suggested to be the ligand for LAG-3, mediating immunosuppressive activities [62]. The “KIEELE” motif within the LAG-3 intracellular domain has been identified as a regulator of LAG3′s inhibitory activity. It interacts with unknown binding partner molecules, transmitting signals that induce cell cycle arrest in T cells and subsequent reduction in T cell expansion [65,66]. Elevated LAG-3 expression has been observed in intratumoral PD-1^+^ T cells and is associated with adverse outcomes in follicular lymphoma [67]. LAG-3 expression correlates with PD-1 expression on TILs, suggesting implications for postoperative recurrence in non-small-cell lung cancer (NSCLC) and compromised anti-tumor responses in gastric cancer [14,68]. Aberrant LAG-3 expression has been identified in various solid tumors, with its expression significantly associated with aggressive tumor progression and clinicopathological characteristics [69]. While the mechanism of immune suppression by LAG-3 remains elusive, evidence of its association with a dysfunctional state in TILs underscores the importance of LAG-3 as a crucial target for immune checkpoint inhibitor (ICI)-based therapy.

#### 2.1.4. T-Cell Immunoglobulin and Mucin Domain-Containing Protein 3 (TIM-3)

TIM-3 is a member of the TIM family, which comprises TIM-1 (encoded by *HAVCR1*), TIM-3 (encoded by *HAVCR2*), and TIM-4 (encoded by *TIMD4*), all of which are conserved in humans [70]. Primarily located on Th1 cells and Type 1 cytotoxic CD8 T cells (Tc1), as well as on Tr1 cells, Tregs, and innate immune cells such as monocytes, DCs, and NK cells, TIM-3 serves as an indicator of T-cell exhaustion, alongside PD-1 [71]. Various ligands, such as galectin-9, phosphatidylserine (PtdSer), high-mobility group protein B1 (HMGB1), and carcinoembryonic antigen cell adhesion molecule 1 (CEACAM-1), bind to TIM-3, interacting with distinct sites on its extracellular IgV domain. Among these, Gal9 and CEACAM-1 binding are associated with the suppression of anti-tumor responses and the dysfunction of TIL subsets [72]. Gal9, located on the tumor cell surface, binds to TIM-3, inducing TIM-3 oligomerization and complex formation with CEACAM1. This initiate signaling cascades that result in immune synapse disruption, ultimately leading to T-cell anergy or apoptosis [73]. The upregulation of TIM-3, especially when combined with PD-1, signifies exhaustion of TILs and is associated with impaired anti-tumor responses across different cancers, including melanoma, NSCLC, and diffuse large B-cell lymphoma (DLBCL) [74,75,76]. High expression of TIM-3 also serves as a prognostic marker in solid tumors, encompassing colon, gastric, cervical, NSCLC, and renal cell carcinoma correlating with unfavorable prognosis and reduced survival rates [70,77]. TIM-3 also delineates a subset of tumor-specific Tregs that are absent in peripheral tissues, which can be effectively targeted for Treg depletion using ICIs [78]. Because of its association with T-cell exhaustion and diminished production of effector cytokines compared to TIM-3-negative counterparts, TIM-3 represents an appealing target for immune checkpoint inhibitor (ICI)-mediated blockade. This strategy aims to rejuvenate anti-tumor responses of T and NK cells [79]. Preclinical studies conducted in mouse models have demonstrated the efficacy of TIM-3 blockade in combination with PD-1 inhibition [73]. Notably, one of these studies emphasized TIM-3′s role as a mechanism of resistance to PD-1 blockade [80]. In summary, TIM-3 emerges as a crucial regulator of T-cell exhaustion and immune response across various cancers, thereby positioning it as a promising target for therapeutic interventions aimed at bolstering anti-tumor immunity.

#### 2.1.5. T-Cell Immunoreceptor with Immunoglobulin and ITIM Domain (TIGIT)

TIGIT has recently emerged as a promising target for immune checkpoint blockade, joining the ranks of other checkpoint inhibitors. It is expressed on activated CD4 and CD8 T cells, NK cells, and Tregs. TIGIT interacts with four ligands: CD111, CD112, CD113, and CD155, with CD155 exhibiting notably higher binding affinity [80]. Expression of CD155 and CD111 is observed on DCs, T and B cells, as well as non-hematopoietic cells, including tumor cells under oncogenic stimulation.

Similar to CTLA-4, TIGIT demonstrates both intrinsic and extrinsic inhibitory activities. Upon binding with TIGIT, CD155 signaling on human monocyte-derived DCs triggers the secretion of the immunosuppressive IL-10, fostering the formation of tolerogenic DCs and consequently inducing an immunosuppressive effect on T cells [81]. In terms of intrinsic signaling, TIGIT, due to its high affinity for CD155, outcompetes DNAM-1 (CD226), thereby inhibiting DNAM1-mediated costimulatory signaling and resulting in reduced IFN-γ production [81,82]. This competition with CD226 also enables TIGIT to suppress the Akt signaling pathway and phosphorylation of FOXO1, consequently attenuating T/NK cell activation, migration, cytotoxicity, and fostering T/NK cell exhaustion [83,84]. A recently identified intrinsic mechanism, independent of CD226, involves TIGIT clustering upon CD155 ligation on TILs. This clustering culminates in the coalescence with the TCR synapse, subsequently leading to the suppression of effector cytokine production [85]. Notably, TIGIT expression is found on exhausted CD8^+^ T cells, serving as a defining marker for T-cell exhaustion [86].

High expression of TIGIT is predictive of tumor progression in multiple myeloma and is associated with poor outcomes and resistance to PD-1 immune checkpoint inhibitors in follicular lymphoma [15]. Furthermore, it is inversely correlated with CD8 T-cell infiltration in cervical cancer. Inhibiting TIGIT/CD155 signaling has shown promise in reversing the exhaustion of CD8^+^ T cells and boosting the effectiveness of anti-tumor treatments in both cervical cancer and multiple myeloma [17,87]. The clinical trial landscape concerning LAG-3, TIGIT, TIM-3, and VISTA indicates that these newly emerged checkpoints are either co-expressed with PD-1 in dysfunctional immune cells or associated with resistance against current PD-1/PD-L1 immune checkpoint inhibitors. Consequently, ongoing trials primarily focus on synergistically blocking one of these checkpoints along with PD-1 or PD-L1 to reinvigorate TILs. Combinatorial approaches are considered optimal to target the compensatory upregulation of immune checkpoints resistant to anti-PD-1/PD-L1 treatments [88,89].

#### 2.1.6. CD39/CD73/A2AR Pathway

In steady-state conditions, extracellular ATP (eATP) levels typically remain low in normal tissue. However, malignancies exhibit an increase in eATP levels due to various factors such as cellular stress, hypoxia, and inflammation. This elevated eATP level activates P2 purinergic receptors expressed by both immune and non-immune cells, triggering a cascade of pathophysiological effects, notably serving as a pro-inflammatory Danger-Associated Molecular Pattern (DAMP) [90]. The accumulated eATP undergoes hydrolysis, initially yielding ADP and AMP, and subsequently transforming into extracellular adenosine (eADO) through the catalytic action of ectonucleotidases CD39 and CD73 [91]. Another pathway, involving the hydrolysis of NAD^+^ by CD38 and CD203, culminates in eADO production by CD73, thus contributing to this process [92]. The signaling of eADO is mediated through adenosine receptors A1R, A2AR, A2BR, and A3R, with A2A and A2B primarily responsible for immunosuppressive signaling [93]. Each element of the adenosine pathway has emerged as a potential immunotherapeutic target in tumors to inhibit eADO formation and signaling.

A2AR, known for its high affinity, is expressed on a variety of immune cells, such as T cells, NK cells, monocytes, macrophages, and DCs. In contrast, A2BR receptor, which exhibits relatively lower affinity, is mainly expressed by macrophages and DCs [94]. Signaling through both A2AR and A2BR involves activation of the PI3K/Akt signaling pathway, subsequently promoting epithelial–mesenchymal transition (EMT) in tumors, tumor cell proliferation, and metastasis [95]. In immune cells, the activation of A2AR and A2BR leads to exhaustion of CD8 T cells and NK cells by suppressing the production of cytotoxic cytokines, including IFN-γ, perforin, and TNFα, through cAMP signaling and inhibition of Lck, an essential kinase for T-cell activation [96,97]. Recent studies have emphasized the upregulation of both receptors in CAR-T cells. Disruption of the A2AR gene or blocking its signaling pathway has been demonstrated to enhance anti-tumor responses by boosting cytotoxicity, proliferation, and preventing exhaustion of CAR-T cells [98,99,100].

CD39 and CD73 are significantly overexpressed on tumor cells and have been associated with adverse outcomes in various cancers. CD73 expression has been implicated in epithelial–mesenchymal transition (EMT), poor prognoses, and resistance to chemotherapy across different cancer types [97,101,102]. Particularly in triple-negative breast cancer (TNBC), CD73 has demonstrated significant clinical significance. Elevated plasma levels of soluble CD73 (sCD73) have emerged as a potential biomarker for anti-PD-1 resistance in melanoma [103]. CD73 plays multiple roles in promoting cell adhesion, migration, invasion, and stemness within cancer cells, irrespective of its enzymatic activity [104].

CD39, encoded by *ENTPD1*, is present on various cell types, including tumor cells, tumor-specific CD4 and CD8 T cells, endothelial cells, fibroblasts, and cells of myeloid lineage [105]. Its expression on Tregs and Tr1 cells correlates with their immunosuppressive function, which may occur independently of co-expression with CD73 [106,107]. Additionally, CD39 expression characterizes populations of exhausted CD8 T cells [108]. Moreover, the expression of CD39 on circulating and intratumoral CD4 and CD8 T cells has been associated with an unfavorable prognosis and resistance to anti-PD1 blockade therapy [107,109]. Its co-expression with CD73 on myeloid-derived suppressor cells (MDSCs) has been shown to be crucial for the immunosuppressive function of MDSCs across various malignancies [110,111,112].

Therefore, almost every component within the adenosine pathway, whether acting independently or in conjunction, exacerbates adverse prognosis, fosters resistance to diverse chemotherapeutic and immunotherapeutic agents, and fuels disease progression. These outcomes predominantly hinge on eADO regulation, orchestrating the modulation of distinct immunosuppressive subsets within the TME. Moreover, recognizing ATP’s immunostimulatory properties highlights the importance of therapeutic interventions aimed at hindering ATP degradation or impeding eADO accumulation within the extracellular matrix (ECM).

#### 2.1.7. V-Domain Immunoglobulin Suppressor of T-Cell Activation (VISTA)

VISTA, known by various aliases including PD-1H, B7-H5, Dies1, Gi24, DD1α, and C10orf54, shares structural similarities with proteins from the B7 and CD28 superfamily. It functions as both a co-inhibitory receptor and ligand, emerging as a novel checkpoint in immune regulation [18,113]. Predominantly expressed on DCs, macrophages, and myeloid cells, VISTA exhibits high expression on naïve T cells, promoting quiescence and tolerance, which diminishes upon activation [114]. Although the intracellular signaling mechanisms of VISTA remain unclear, it has reported ligands such as PSGL-1, Syndecan-2, LRIG-1, VSIG8, and VSIG3, with the latter two confirmed to engage in immune suppression [115,116].

Human PSGL-1, expressed widely across leukocytes, albeit with lower levels in B cells, interacts with VISTA under lower pH conditions, often observed in the TME, resulting in the suppression of T-cell function [117]. Furthermore, it can induce T-cell exhaustion independently of VISTA [118]. Conversely, the binding of VSIG3 and VSIG8 to VISTA significantly reduces cytokine and chemokine production by human T cells, including IFN-γ, IL-2, IL-17, CCL5/RANTES, CCL3/MIP-1α, and CXCL11/I-TAC [119,120]. Analysis of VISTA expression across 31 different malignant tumor types using TCGA pan-cancer samples reveals elevated normalized VISTA expression in mesothelioma, low-grade glioma (LGG), kidney renal clear-cell carcinoma (KIRC), pancreatic adenocarcinoma (PAAD), head and neck squamous cell carcinoma (HNSCC), sarcoma, and glioblastoma (GBM) [116,121]. Increased VISTA expression is observed on tumor-infiltrating macrophages, MDSCs, and tumor cells, with some exhibiting resistance to CTLA-4 and PD-1 blockade therapy. Preclinical studies in murine models demonstrate efficacy in reducing Treg cell differentiation and enhancing T-cell effector function when VISTA is co-inhibited with PD-1 or PD-L1 [116,122,123].

#### 2.1.8. B7-H3/B7-H4

Both B7-H3 (CD276) and B7-H4 belong to the B7 transmembrane protein family, alongside PD-L2 (B7-DC), PD-L1 (B7-H1), and B7-H2, a co-stimulatory ligand. While the receptors for B7-H3 remain unclear, evidence suggests TREM-like transcript 2 (TLT-2) as a potential receptor, expressed on T cells and NK cells [124,125]. There may exist other yet-to-be-discovered receptors for B7-H3 on immune cells. As for B7-H4, its receptor, initially identified as BTLA (CD244), is still undetermined [126].

Although in mouse models B7-H3 expression on tumor cells has demonstrated NK/CD8-mediated tumor regression, such evidence is seldom found in humans. Instead, clinical and preclinical data demonstrate an association between CD276 expression and poor prognosis, reduced TILs infiltration, impaired anti-tumor responses by CD8^+^ T cells and NK cells, as well as heightened metastasis across several cancer types, such as pancreatic, esophageal, and cervical cancer [127,128,129,130]. In NSCLC, it is also associated with active Treg infiltration [131]. Several preclinical studies have demonstrated the beneficial effects of B7-H3 blockade, as evidenced by reduced tumor growth, decreased metastatic potential, and improved survival rates [132]. Other predicted mechanisms include JAK/STAT3-mediated production of IL-10 and TGFβ [133], promotion of switching from M1 to M2 macrophages [134], and suppression of anti-tumor immunity via the CCL2-CCR2-M2 macrophage axis [135]. Given its diverse pro-tumorigenic and anti-immunogenic roles, the complex functions of B7-H3 still require further exploration. Given its wide expression on tumor cells and its association with aggressive disease and metastasis, various B7-H3-targeted ADCs, CART therapies, and mAbs for ADCC are presently under evaluation [136].

Similar to B7-H3, B7-H4 is expressed on both tumor cells and TAMs, with high expression correlating with tumor aggressiveness and diminished survival [137,138,139]. Various studies have proposed mechanisms of immunosuppression mediated by B7-H4. In ovarian cancer, the induction of B7-H4 on macrophages has been shown to be regulated by Tregs, and blocking B7-H4 on macrophages restored their capacity to stimulate T cells [140]. Additionally, another study demonstrated that antigen-TCR-specific T cells could be inhibited by B7-H4, whether it is expressed in cis on APCs or tumor cells or in trans on TAMs [141]. In murine models of TNBC, B7-H4 degradation leads to enhanced IFN-γ production by CD8 T cells and increased DC-mediated phagocytosis of tumor cells. Others have demonstrated that B7-H4 interrupts initial TCR activation-related events, ultimately leading to the suppression of cytokine production by T cells [142]. Several ongoing trials are also focusing on targeting B7-H4 as a tumor-specific antigen to induce cell death. The utilization of a B7-H4-targeted ADC has exhibited anti-tumor efficacy in advanced solid tumors. An ADC directed against B7-H4, employing a vedotin linker and monomethyl auristatin E payload, demonstrated ADC-mediated cytotoxicity and induced cell death through ADCC and ADCP mechanisms in murine models of ovarian and breast cancer. This ADC is currently undergoing a Phase I trial [NCT05194072] [143]. Overall, both B7-H3 and B7-H4 are emerging as targets for immunotherapy through various approaches.

#### 2.1.9. SIRPA/CD47

The CD47 signaling system within healthy tissue operates as a mechanism to evade phagocytosis by transmitting a “don’t eat me” anti-phagocytic signal to macrophages. CD47, a member of the immunoglobulin superfamily, is predominantly expressed on cells of the myeloid lineage, such as macrophages, monocytes, DCs, and granulocytes [144]. Tumor cells upregulate CD47 expression to evade phagocytosis, with CD47 acting as a ligand for three different receptors: signal regulatory protein alpha (SIRPα), thrombospondin-1 (TSP-1), and integrins, including αvβ3 and α2β1. SIRPα is a transmembrane protein expressed on all myeloid cells, including monocytes, macrophages, and neutrophils and primarily CD47 is the ligand for SIRPα. Engagement of CD47 with SIRPα initiates phosphorylation of intracellular C-terminal ITIM motifs and triggers anti-inflammatory signaling in phagocytes, thus reducing phagocytosis [145]. Inhibition of the CD47-SIRPα interaction by antibodies has shown increased phagocytosis of tumor cells and reduction of tumor burden across various cancer types [145,146]. High CD47 expression is associated with poor prognosis, increased epithelial–mesenchymal transition (EMT), tumor invasiveness, and lower response to ICIs in different solid tumor types including oral squamous cell carcinoma, nasopharyngeal carcinoma, TNBC, and NSCLC [147,148,149,150,151,152].

A thorough analysis of the correlation between CD47 and other immune checkpoints showed that CD47 expression was positively associated with elevated expression of key immune checkpoints across most cancer types, as well as with tumor mutational burden (TMB) in certain solid cancers such as melanoma, esophageal, and bladder cancer [147]. This indicates the importance of synergistic blockade of SIRPα-CD47 signaling and immune checkpoints like CTLA-4, PD-1, and PD-L1. Numerous studies have highlighted the effectiveness of dual blockade, encompassing DNA sensing, DC cross-presentation, augmentation in stem-like progenitor CD8 T cells, thereby leading to an enhanced anti-tumor response [153,154,155,156]. Therefore, considering the ubiquitous expression of CD47 across various tumors and its pivotal role in immune suppression via the SIRPα-CD47 signaling pathway, it strongly advocates for CD47 as a potent target for immune checkpoint blockade (ICB), either as a standalone approach or in conjunction with other ICI agents.

#### 2.1.10. NKG2A/HLA-E/CD94

Natural killer group protein 2A (NKG2A), also referred to as KLRC1, is an inhibitory receptor containing immunoreceptor tyrosine-based inhibitory motifs (ITIMs), expressed on circulating T and NK cells [157]. Its presence on T cells can be augmented following T-cell receptor (TCR) stimulation and further enhanced by cytokine stimulation, such as IL-15 and TGF-β [158,159]. NKG2A forms a heterodimer with CD94 on the cell surface. When it binds to its ligand, the major histocompatibility complex class I (MHC-I) molecule HLA-E in humans, it inhibits the functions of both T cells and NK cells, thereby serving as an immune checkpoint regulator for these cell types [160,161,162]. Infiltration of TILs expressing NKG2A-CD94^+^ correlates with poorer survival outcomes in colorectal cancer, ovarian cancer, and cervical carcinoma [163,164,165]. In preclinical models, the efficacy of NKG2A blockade was demonstrated with an improved clinical response to cancer vaccines [166]. HLA-E, highly expressed in tumor samples, contributes to immune suppression through the NKG2A-CD94 pathway. Increased expression of HLA-E in tumors is associated with reduced survival rates and resistance to CD8 cytotoxic activities [167,168]. However, targeting NKG2A is preferred over HLA-E blockade, as blocking HLA-E would also interfere with the interaction between HLA-E and the activating receptor NKG2C on NK cells.

#### 2.1.11. LILRB1/LILRB2: HLA-G

The leukocyte immunoglobulin-like receptors (LILRs) belong to the paired receptor superfamily, consisting of inhibitory LILRBs (LILRB1-5) and LILRAs (LILRA1-2). LILRB1 and 2 possess extracellular immunoglobulin-like domains with cytoplasmic ITIM-like motifs. These receptors recognize both classic MHC class I (HLA-A, HLA-B, and HLA-C) and non-classic MHC class I (HLA-E, HLA-F, and HLA-G) molecules in cis and trans configurations and are expressed on macrophages, DCs, B cells, certain T-cell subsets, and malignant cells [169,170]. LILRB-mediated immunosuppression is primarily observed in association with the binding partner HLA-G, recognized as an immune checkpoint [171]. Interaction between HLA-G on tumor cells and LILRB1-expressing NK cells and CD8^+^ T cells inhibits cytotoxicity through the ITIM/SHP-2-mediated pathway. LILRB1 on naïve T cells promotes their differentiation into Th2 cells and Tregs. In clear-cell renal-cell carcinoma (ccRCC), LILRB1^+^ TIL CD8 T cells exhibit reduced cytotoxicity in the presence of HLA-G-expressing target cells, which can be reversed by anti-HLA-G-mediated blockade [172]. Blocking LILRB1 on NK cells enhances tumoricidal activities against various cancers both in vivo and in vitro [173]. LILRB2 blockade also induces the reprogramming of TAMs from NSCLC cancer patients into an inflammatory M1-like phenotype [174]. Expression of the MHC class I component β2-microglobulin (β2M) on tumor cells correlates positively with resistance to phagocytosis. Blockade of LILRB1 or LILRB2 on macrophages induces phagocytosis of tumor cells [175]. Therefore, targeting HLA-G/LILRB pathways with antibodies, alone or in combination with other ICIs, might constitute a promising strategy for breaking down tolerance in tumors and promoting rejuvenation of exhausted tumor-infiltrating immune cells.

#### 2.1.12. Sialic Acid-Binding Immunoglobulin-Type Lectins (Siglecs)

Siglecs, members of the type 1 immunoglobulin-like family, encompass both activating and inhibitory members, including Siglec-3, Siglec-5, Siglec-7, Siglec-9, and Siglec-10, each equipped with intracellular ITIM-like domains that transmit inhibitory signals by recruiting SHP1/SHP2 [176,177]. These receptors specifically recognize sialic acid-containing glycans. Numerous studies have implicated sialic acid in fostering a myeloid pro-tumorogenic phenotype, underscoring the pivotal role of Siglecs as crucial checkpoints for immunotherapeutic targeting [178,179,180,181]. Among them, Siglec-7, Siglec-9, and Siglec-10 are part of the CD33 family and are prominently expressed on myeloid lineage cells, such as TAMs, NK cells, and certain intratumoral T-cell subsets [179,182]. Conversely, Siglec-15 exhibits predominant expression on intratumoral macrophages and displays immunosuppressive characteristics [181,183]. Additionally, Siglec-10 has been shown to interact with sialylated CD24, leading to the suppression of tumor cell phagocytosis by macrophages [184,185].

Furthermore, Siglecs exert influence over T-cell and NK-cell functionalities, with Siglec-9 engagement on CD8 T cells and Siglec-7/9 on NK cells leading to diminished effector function [182,186,187]. Targeting the interaction between sialic acids present in tumor cells and Siglec receptors expressed on immune cells is emerging as a promising avenue in anticancer strategy. Various approaches are being explored, including the use of sialic acid mimetics, sialidases, blocking antibodies against Siglecs, and CAR-T cells directed towards sialylated antigens [188]. Combination therapies that integrate Siglec pathway inhibitors with anti-GD2 and anti-CD47, or radiotherapy, demonstrate potential in overcoming resistance to existing therapies [189,190]. Ongoing clinical trials are investigating Siglec-10 inhibitors (NCT06219499), bispecific antibodies targeting CD24 and CD3, and CART therapy involving Siglec signaling inhibitors alongside bispecific antibodies targeting CD24 and CD3 (ONC 783). Given the promising targets within the Siglec family, these approaches hold significant potential for enhancing cancer treatment outcomes.

### 2.2. Metabolic Reprogramming to Foster an Immunosuppressive Environment

Metabolic reprogramming, characterized by the alteration of metabolic pathways, significantly contributes to fueling the proliferation of tumor cells, providing them a progressive advantage over non-malignant tissues. The pivotal mutations in tumors drive neoplastic cells to adopt metabolic programs that not only facilitate proliferation but also endow them with mechanisms to evade immune surveillance. This phenomenon establishes metabolic reprogramming as a hallmark of cancer [191,192]. Metabolic reprogramming in cancer cells exhibits several notable features. Firstly, it involves increased glucose consumption through aerobic glycolysis, leading to the accumulation of end products such as lactate—a phenomenon famously described as the Warburg effect. Secondly, cancer cells maintain a hypoxic environment due to their elevated metabolic rate and the disorganized vasculature within the TME. Thirdly, amino acids like glutamine, arginine, and tryptophan play pivotal roles in driving tumor progression. Fourthly, tumor cells upregulate fatty acid metabolism, resulting in an increased production of fatty acid metabolites. Interestingly, the TME, shaped by these metabolic alterations, presents a formidable challenge for immune cells infiltrating tumors, consequently impacting overall anti-tumor immunity [192,193,194,195]. Various types and subsets of immune cells, each with distinct nutrient requirements for metabolic programming, thus confront unique challenges upon their migration into the TME.

Similar to cancer cells, T cells exhibit a preference for aerobic glycolysis over oxidative phosphorylation (OXPHOS) to generate ATP. While glucose uptake and glycolysis are dispensable for T-cell proliferation and survival, they are essential for effector cytokine IFN-γ production [196,197]. In the glucose-limited TME, cancer cells compete with immune cells for glucose uptake, posing a serious threat to the function of tumor-infiltrating immune cells. Tumors metabolically restrict T-cell and NK-cell function by consuming glucose and limiting glycolysis, which suppresses mTOR activity, IFN-γ production, and anti-tumor immunity. This metabolic modulation in T cells has been demonstrated to be linked with PD-1 [198]. Excessive glycolysis under hypoxic conditions leads to the accumulation of the metabolic byproduct lactate in the TME. Lactate, produced abundantly by tumor cells, exerts profound immunosuppressive effects by impeding the proliferation of effector T cells and NK cells, inhibiting IFN-γ production in CTLs, and promoting Treg survival in a Foxp3-dependent manner within a lactate-rich, glucose-depleted TME [199,200,201]. Within the myeloid compartment, lactate promotes M2 polarization through increased arginase and HIF1α expression, facilitates the generation of MDSCs, and inhibits monocyte activation and DC differentiation [202]. The accumulation of lactate contributes to lowering the pH of the TME, thereby intensifying its immunosuppressive properties. Findings reveal that VISTA, an immune checkpoint, interacts with its receptor PGSL1 to selectively suppress T cells in an acidic pH environment [117].

Dysregulated vasculature within the TME establishes a hypoxic environment, inducing the stabilization of hypoxia-inducible factor genes (HIF). HIF, in turn, promotes the transcription of glycolysis-associated genes and facilitates angiogenesis. The impact of hypoxia and HIFs on T cells is multifaceted. While certain studies underscore the crucial role of hypoxia and HIF1α in enhancing cytolytic function and anti-tumor response in CD8 T cells, they have also been associated with immunosuppression [203,204]. Prolonged activation under hypoxic conditions induces exhaustion in CD8 T cells by triggering mitochondrial stress [205]. Furthermore, the hypoxic environment leads to HIF1α-mediated transcription of Foxp3, contributing to an abundance of Treg cells [206]. In colorectal cancer patients, regions of intense hypoxia in the TME were found to correlate with high VISTA expression on MDSCs and were associated with poor survival [207]. It is essential to highlight the contextual differences in the influence of lactate and hypoxia on T-cell effector function. The combined effects of a hypoxic environment and an acidic state associated with lactate can lead to mitochondrial stress, ultimately resulting in T-cell exhaustion. This contrasts with the lactate-dependent increase in effector CD8 T cells observed in non-malignant tissues.

Amino acid metabolism plays a crucial role in regulating TME. Both tumor cells and immune cells heavily depend on amino acids such as arginine, serine, and tryptophan for proliferation. Specifically, glutamine, tryptophan, and tyrosine are crucial amino acids associated with tumor progression. The limited availability of these amino acids instigates a competitive dynamic among cancer cells, pro-tumorigenic TAMs, and T cells. Glutamine emerges as a critical nutrient for cancer cells, supporting their metabolism and proliferation while also exerting immunosuppressive effects by influencing myeloid cells, notably by fueling M2 macrophages and MDSCs. Inhibiting glutamine metabolism in breast cancer has demonstrated a shift toward inflammatory M1 macrophages and reduced tumor progression, achieved by diminishing MDSC recruitment [208,209]. Targeting glutamine metabolism, particularly through the inhibition of glutaminase (GLS) in CD4 T cells, leads to differential regulation of proliferation, differentiation, and effector functions. Pan glutamine blockade using 6-diazo-5-oxo-l-norleucine (DON), either alone or in combination with anti-PD-1 therapy, or even through GLS-specific inhibition during CD8 T-cell activation, has demonstrated enhancement in CD8 cytotoxicity and overall anti-tumor activity [210]. Additionally, it has shown improvements in CD8 proliferation and the persistence of tumor-specific CD8 cells in adoptive T therapy [211]. The depletion of extracellular arginine by arginase (ARG)-expressing MDSCs suppresses anti-tumor responses. Conversely, inhibiting ARG activity on MDSCs enhances T-cell anti-tumor responses [212,213]. Arginine depletion in NK cells hinders their proliferation and IFN-γ production induced by IL-12/IL-18 [214]. Tryptophan, serving as a substrate in the kynurenine pathway, undergoes regulation by enzymes such as indoleamine-2,3-dioxygenase (IDO1), IDO2, and tryptophan-2,3-dioxygenase (TDO). Increased IDO1 activity prevents effector T-cell activation, dampens NK-cell function, facilitates Treg activation, and promotes the expansion and activation of DCs and MDSCs [215]. Targeting amino acid metabolism to improve anti-tumor function is contingent on context and target specificity due to the dual reliance of tumor cells and immune cells on these amino acids within the TME.

Cancer cells exhibit a heightened avidity for lipid and cholesterol droplets, which is satisfied by upregulating enzymes involved in lipid and cholesterol biosynthesis, along with an increased uptake of exogenous lipids. Accumulation of fatty acids in myeloid cells enhances oxidative metabolism and promotes their immunosuppressive function. Excessive unsaturated fatty acids can polarize myeloid cells into immunosuppressive M2-like TAMs, while lipid accumulation can hinder antigen presentation by tumor-infiltrating DCs, resulting in inadequate T-cell priming [216,217,218]. Simultaneous blockade of lipid transporters inhibits tumor progression as well as M2 macrophage polarization. In T cells, while fatty acid oxidation (FAO) is crucial for memory CD8 T-cell formation, it also promotes the differentiation of immunosuppressive Tregs [219]. In melanoma models, CD8^+^ TILs increase FAO to sustain effector functions, yet the lipid transporter CD36 enhances Treg survival [220]. This underscores the specificity of different fatty acid pathways in determining immune cell fate and function, alongside their independent effects on tumor cells. While several key functional attributes influence metabolic programs in tumors, the heterogeneity of the TME and the various stages of tumor progression play a pivotal role in shaping the evolution of the TME’s metabolic properties. Consequently, therapeutic targeting of a single metabolic component can have diverse effects on both tumor cells and immune components.

### 2.3. Recruitment of Immunosuppressive Cells into the Environment

Cancer-associated fibroblasts (CAFs), along with cells of myeloid lineage such as DCs, MDSCs, and neutrophils, play crucial roles in inducing immunosuppression within the TME. Among the three main types of CAFs—myofibroblastic (MyCAF), inflammatory (iCAF), and antigen-presenting CAFs (apCAFs)—iCAFs impede immune cell infiltration, induce TIL exhaustion, and confer resistance to immune checkpoint blockade (ICB) by secreting IL-6, CXCL12, CCL2, and IL-1 [221,222]. The apCAF subset expresses MHC II without costimulatory molecules and is implicated in Treg induction by activating CD4 T cells in pancreatic cancer [223]. One of the hallmarks of immune-excluded tumors is the presence of tumors enveloped by a network of matrix fibers containing myCAFs and CD8 T cells, where CD8 cells in the stromal matrix fail to infiltrate into the tumor [224,225]. However, precise regulatory mechanisms underlying CD8 T-cell function by myCAFs remain poorly understood.

Within the myeloid-derived cell compartment, diverse mononuclear subsets coexist, including TAMs, MDSCs, and DCs, alongside polymorphonuclear cells such as neutrophils and mast cells. Inflammatory cues associated with cancer, such as IL6, IL4, IL13, and TNF, promote the generation and expansion of heterogeneous immature myeloid-derived cells, comprising polymorphonuclear/granulocytic (PMN/G-MDSCs) and monocytic (M-MDSCs) subsets. These cues also facilitate their recruitment through signaling pathways involving IL8, CCL2, and CXCL12 [226]. M-MDSCs are characterized by the presence of CD11b^+^CD14^+^CD33^+^HLA^−^DR^low/−^ markers, whereas PMN/G-MDSCs express CD11b^+^CD15^+^HLA^−^DR^low^CD66b^+^ [227]. However, the overlap in markers complicates the accurate delineation of neutrophil and MDSC subsets. Various mechanisms, such as HIF1a induction, promote ENTPD2/CD39L1 expression on MDSCs, hindering further differentiation in the TME [228]. Additionally, chemokine signaling via CCL2, CXCL12, and CXCL18/IL8 is associated with MDSC accumulation and adverse outcomes in ovarian and colorectal cancers [229,230]. The immunoregulatory functions of MDSCs have been delineated in murine models across various cancer types, including the induction of de novo generation of FoxP3^+^ Treg cells, the polarization of macrophages towards an immunosuppressive M2 phenotype, and the production of ROS to inhibit IFN-γ secretion and proliferation of antigen-specific CD8^+^ T cells [231,232]. Furthermore, MDSCs also impair NK-cell function through nitric oxide production, leading to a reduced response to mAb therapy [233]. Additionally, MDSCs expressing ectonucleotidases CD73 and CD39, along with the immune checkpoint PD-L1, contribute to immunosuppression in the TME and resistance to immune checkpoint blockade therapy [234,235].

While DCs play a crucial role in initiating T-cell responses against tumors, a subset within tumors adopts a tolerogenic role, promoting immune suppression. Human DCs are categorized into classical DCs (cDCs), which include cross-presenting CD8α^+^ or CD103^+^cDC1 cells that present antigens to CD8 T cells, CD11c^+^cDCs, and plasmacytoid DCs (pDCs). In addition to immune checkpoint-mediated T cell suppression, tumors impair DC function through strategies such as hindering cDC recruitment and differentiation and impeding antigen presentation [236]. Tumors expressing β-catenin suppress the production of CCL4, thereby limiting DC infiltration, while IL-6 from immune and cancer cells inhibits cDC differentiation [237]. In tumors, infiltrating NK cells can produce CCL5 and XCL1 to promote DC recruitment and secrete FLT3 for DC maintenance, yet this ability is suppressed by tumor-produced PGE2 and VEGF [238,239]. High Mobility Group Box 1 (HMGB1) on DCs aids in the innate sensing of nucleic acids from dead cells and antigen presentation, but its interaction with highly expressed TIM3 on T cells disrupts this process [240]. The accumulation of tumor-derived lipid by-products in tumor-associated DCs due to endoplasmic reticulum stress impairs antigen processing and T-cell activation [241]. Tumor-infiltrating pDCs exacerbate T-cell dysfunction by promoting regulatory T-cell expansion in an ICOSL-dependent manner, which correlates with a poor prognosis [242].

Among the two types of Tregs—inducible (iTregs) and natural—tumor-specific Tregs are primarily of the inducible type. Tregs secrete inhibitory cytokines like IL-10, TGF-β, and IL-35 to suppress the cytotoxicity of tumor-specific CD8 T cells, hamper antigen presentation by DCs, and hinder CD4 helper T-cell function. IL-35, secreted by Tregs, also stimulates CD39^+^Tregs, further enhancing immunosuppressive function by recruiting MDSCs [243,244]. In claudin-low tumors, despite high TIL infiltration, ICI blockade fails to revive T-cell anti-tumor responses due to the elevated proportion of Treg cells in the tumor. Consequently, only Treg depletion demonstrates the benefits of ICI treatment [245]. A novel IL-33/ST2 signaling-based activity of Tregs has been identified, showing increased production of immunosuppressive IL-10 and TGF-β, and decreased T-cell proliferation in HNSCC [246]. Tregs can inhibit CD8^+^ T-cell and DC function through membrane-bound TGF-β, thereby modulating the body’s anti-tumor immune response. Additionally, they disrupt T-cell metabolism by inducing IL-2-mediated oxidative stress and altering amino acid metabolism. Tregs also express enzymes like IDO and CD39/CD73, which generate immunosuppressive metabolites [247].

TAMs are broadly classified into M1 macrophages, induced by bacterial products and pro-inflammatory cytokines, which demonstrate anti-tumor activity, and M2 macrophages, induced by immunoregulatory cytokines, promoting tumor growth by secreting tissue-remodeling and pro-angiogenic factors. However, the intricate nature of TAMs challenges simple M1/M2 classification, leading researchers to propose alternative categories such as M2a, M2b, and M2c, or to adopt terms like M1-like and M2-like. Nevertheless, these classifications may still inadequately capture the full spectrum of TAM heterogeneity, as TAM subsets can co-express genes associated with both M1 and M2 activation and express genes beyond the classical M2 phenotype [248,249]. In addition to their prominent role in tumor progression, which involves facilitating angiogenesis, inducing tumor cell proliferation and migration, and aiding in metastasis, TAMs also play crucial roles in immunosuppression [250]. Furthermore, M2 macrophages contribute to tumor progression by remodeling the TME through the release of matrix metalloproteinases (MMPs) such as MMP2, 7, 9, and 12, inducing angiogenesis via VEGF secretion, and recruiting Tregs through CCL2 production [251].

### 2.4. Antigen Presentation

The antigen presentation process is crucial for maintaining immune responses and immune surveillance against cancer. Immunosuppression involves modulating the antigen presentation machinery (APM), which is manifested through alterations in MHC genes and APM components, as well as immunopeptidome modulation to evade antigen presentation [252,253]. The collection of peptides, typically 8–16 amino acids long, presented on class I and II MHC molecules is known as the immunopeptidome. Within malignant tissue, novel epitopes emerge through mechanisms such as somatic missense mutations, insertions, deletions, frameshifts, nonsense mutations, and splice site mutations. These mechanisms aid in distinguishing between malignant and healthy tissue-derived cells. The tumor mutational burden, while part of immune evasion mechanisms, plays a crucial role in determining sensitivity to ICI treatment in NSCLC, melanoma, gastrointestinal cancers, and autologous T cell-based therapy in melanoma [254,255,256].

DCs prime CD8 T cells in the draining lymph nodes via peptide-MHCI-based antigen presentation. MHCI, encoded by HLA-A/B/C genes, exhibits antigenic preferences that impact tumor antigen presentation and the efficacy of therapeutic responses to ICIs [32]. Moreover, heterozygosity at all three HLA-I loci and HLA evolutionary diversity (HED) independently contribute to enhancing clinical outcomes in response to ICB therapy in NSCLC, gastrointestinal cancer, and renal cell carcinoma [257,258,259]. Mechanisms such as somatic loss of heterozygosity (LOH) of HLA genes and somatic mutations in HLA-I genes are identified as mechanisms of immune evasion [260]. Specific HLA-I alleles and supertypes are associated with the likelihood of benefiting from ICIs. For instance, in melanoma, the HLA-B44 supertype is associated with improvement, whereas in NSCLC, HLA-B62 and HLA-A*03 in the bladder and renal cancer are associated with inferior overall survival (OS) [261,262]. A robust correlation between HLA LOH, neoantigen burden, and PD-L1 expression has been observed across various tumor types, with the highest LOH noted in HNSCC [263].

HLA expression is regulated at multiple levels, including transcriptional, post-transcriptional, and post-translational regulation. IFN-γ signaling via the JAK/STAT pathway and transcription factors such as IRF1, NLRC5, and NF-κB p65 induce transcription of HLA and other components of the APM machinery like β2M [264]. Reduced NLRC5 has been shown to impair HLA-I expression, reduce CD8^+^ T-cell activation, and is linked with inferior OS outcomes [265]. Conversely, several miRNAs, such as miR-148a-3p, miR-125a-5p, and miR-27a, suppress MHC I expression in tumor cells, inhibiting the cytotoxic anti-tumor activity of T cells and showing an association with poor prognosis in different cancer types [266,267]. Microdeletions and/or insertions in the repetitive nucleotide motifs of β2M, along with mutations on β2M, have strong associations with MSI in patients with colorectal cancer and melanoma, linking the presence of a high neoantigen burden with immune escape through altered antigen presentation [268].

Processes such as autophagy, ER-associated degradation, and endocytosis promote HLA protein degradation or removal from the cell surface, compromising antigen presentation to CD8 T cells [252]. NBR1-mediated selective autophagy and ER-mediated degradation of HLA I promote immune evasion in pancreatic cancer [269]. Immune evasion is also observed through suppressed antigen presentation by endocytosis of the peptide-MHC complex in breast cancer [270]. In NSCLC, diminished expression of immunoproteasome subunits β1i, β2i, and β5i is linked to a mesenchymal phenotype and results in a lower abundance of MHCI-bound peptide [271]. Conversely, heightened expression of immunoproteasome genes *PSMB8* and *PSMB9* is associated with enhanced OS, increased immune cell infiltration, and serves as a predictor of positive responses to ICIs [272]. Epigenetic mechanisms, such as DNA methylation and histone modifications, exert control over APM genes, including HLA. Hypermethylation and repressive histone marks like H3K27me3, facilitated by PRC2, reduce HLA expression in cancer, thereby impairing antigen presentation and anti-tumor immune responses. Mutations in PRC2 components like EZH2 have also been implicated in HCC as a negative prognostic marker [273]. Apart from MHCI, preclinical studies have highlighted the role of MHCII-based neoantigen presentation to CD4 in shaping responses to immunotherapy [274]. The size of the immunogenic mutational burden presented by MHC II predicts responsiveness to ICIs in patients with NSCLC or melanoma, and MHCII-restricted neoantigens can be non-overlapping with MHCI-restricted neoepitopes [275].

## 3. Cancer Immunotherapeutic Strategies

**Figure 1:**
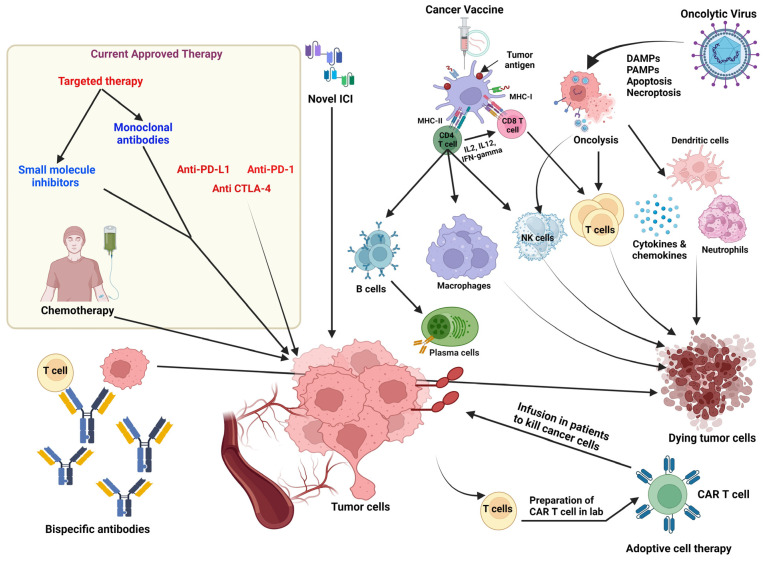
Figure illustrates various cancer immunotherapy strategies, featuring traditional approaches highlighted in yellow, alongside emerging immunotherapeutic approaches which include adoptive cell therapy, cancer vaccines, CAR T-cell therapy, bispecific antibodies, and oncolytic viruses (created with BioRender.com; accessed on 30 March 2024).

Therapeutic approaches are primarily determined by the immunogenicity and immunophenotype of the tumor. These approaches are guided by a combination of factors discussed in the preceding sections. Immune checkpoint inhibitors, for instance, can serve a dual role: converting a “cold” tumor into a “hot” one to attract more immune cell infiltration and providing a boost to T cells undergoing exhaustion [276,277]. Other therapeutic strategies, such as oncolytic viruses or cancer vaccines used in combination with radiotherapy and/or chemotherapy, can enhance tumor antigen presentation and aid in boosting T-cell priming (Figure 1). In the following discussion, we will delve into the major immunotherapeutic strategies and the key determinants of success in these approaches.

### 3.1. Antibody-Based Therapy

Since the approval of rituximab (anti-CD20) in 1997, antibody-based oncology therapeutics have gained prominence. Immunoglobulins have emerged as a crucial immunotherapeutic approach owing to their specificity, cellular targeting, and killing potential, as well as their prolonged serum life. These therapeutics operate by blocking receptor–ligand interactions to enhance immune cell function, tagging cells for immune-mediated killing mechanisms, and delivering cytotoxic payloads for direct tumor cell killing [278]. Among the primary subtypes of human antibodies, the IgG isotype and its related antibody fragments IgG1 and IgG4 are the most frequently utilized for therapeutic applications. Ongoing efforts to optimize monoclonal antibody-based therapeutics focus on improving target affinity enhancing effector function reducing immunogenicity, and extending half-serum life [279,280].

#### 3.1.1. Immune Checkpoint Blockade (ICB)

Although immune checkpoints are the primary regulators of immune tolerance, tumors often exploit them to evade immune surveillance. Since the approval of the first immune checkpoint inhibitor (ICI), ipilimumab (anti-CTLA-4), in 2011, additional ICIs have emerged as a significant therapeutic approach to reinvigorate anti-tumor responses and eliminate malignant cells. Following ipilimumab’s approval, several ICIs targeting PD-1 and PD-L1 have been approved for treating melanoma, NSCLC, and renal cell carcinoma [281]. Currently, there are six approved monoclonal antibody (mAb)-based therapies targeting the PD-1/PD-L1 axis. Anti-PD-1 antibodies pembrolizumab, nivolumab, and cemiplimab are approved for melanoma, lung, renal cell, and head and neck cancers, and anti-PD-L1 antibodies atelizumab, avelumab, durvalumab are approved against lung, Merkel cell carcinoma (MCC), and urothelial cancers [282].

Combination therapy involving ipilimumab and nivolumab or pembrolizumab, which dual-target PD-1/PD-L1 and CTLA-4, is now a standard regimen for achieving an effective and durable immune response [283]. The Phase III CHECK-067 trial (NCT01844505) reported the longest median OS and demonstrated durable, improved clinical outcomes with nivolumab plus ipilimumab or nivolumab alone versus ipilimumab in patients with advanced melanoma [284]. Ipilimumab and nivolumab remain the only FDA-approved dual-checkpoint-based immunotherapy for intermediate to poor-risk metastatic renal cell carcinoma (mRCC) and have replaced small-molecule-based RTK inhibitor sunitinib as the standard care for mRCC [285]. PD-1/PD-L1-targeted ICIs are also included in treatment regimens in combination with chemotherapy and radiotherapy. Adding pembrolizumab (aPD-1) to the standard chemotherapy regimen for previously untreated metastatic non-squamous NSCLC improved OS and progression-free survival (PFS) and has been approved by the FDA as the first-line treatment for advanced non-squamous NSCLC [286]. The anti-PD-L1 agent atezolizumab, in combination with various chemotherapy regimens, has been FDA-approved for triple-negative breast cancer (TNBC), small-cell lung cancer (SCLC), and non-squamous NSCLC [287,288,289]. The Phase III trial findings (NCT02125461) demonstrated that durvalumab, a PD-L1 inhibitor, has established itself as the preferred standard of care for patients with unresectable stage III NSCLC, who do not show any disease progression following chemoradiotherapy [290].

All the newly emerged checkpoints LAG3, TIM3, TIGIT, or VISTA have been found to be either co-expressed with PD-1 in dysfunctional immune cells or associated with resistance to existing PD-1/PD-L1 ICIs [89]. Thus, most of the current trials are focusing on the synergistic blockade of one of these checkpoints along with PD-1 or PD-L1 or even CTLA-4. As most of these ICI agents aim towards reinvigorating TILs, combinatorial approaches are the best to target these compensatory upregulated immune checkpoints that resist anti-PD-1/PD-L1 treatment. In June 2022, a dual immunotherapy regimen, relatlimab–nivolumab fixed-dose combination (Opdualag), received accelerated FDA approval for treating metastatic melanoma. This marks the first FDA-approved anti-LAG-3 mAb combination therapy, positioning LAG-3 as the third clinical ICI target in the clinic, alongside PD-1/PD-L1 and CTLA-4 (NCT03470922) [291]. While approved in conjunction with nivolumab, the relatlimab–nivolumab regimen demonstrates a lower incidence of side effects compared to the nivolumab–ipilimumab regimen. Additionally, research is underway to assess a combination therapy involving domvanalimab, a humanized IgG1 mAb for TIGIT, alongside zimberelimab (aPD-1) [NCT04736173, and NCT05502237] for PD-L1-high NSCLC and with durvalumab [NCT05211895] for lung cancer. A study is investigating the efficacy and safety of the anti-PD-1 antibody retifanlimab combined with INCAGN02385 (anti-LAG-3) and INCAGN02390 (anti-TIM-3) antibodies as the first-line treatment in PD-L1^+^ recurrent or metastatic SCCHN (NCT05287113). A clinical trial with anti-TIM3 (sabatolimab) in combination with anti-PD-1 (spartalizumab) has shown anti-tumor activity (NCT02608268) [292]. Several clinical trials are using TIM-3 antibodies, including Sym023 (NCT033114112), ICAGN02390 (NCT03652077), TSR-022 (NCT02817633), MBG453 (NCT02608268), LY3321367 (NCT03099109), BGBA425 (NCT03744468), R07121661 (NCT03708328), and BMS-986258 (NCT03446040) [293]. Moreover, clinical trials (Phase I, NCT04669899) are examining a humanized anti-LILRb1 either alone or in combination with pembrolizumab for treating advanced refractory solid tumors. Trials involving the CD47 antibody magrolimab include phase 1 PNOC025 (NCT05169944) for progressive brain tumors and Phase 2 (NCT04827576) for NSCLC and urothelial carcinoma (UC). However, all other AML studies, including the Phase III study (NCT05079230), have been suspended due to increased risks of primary infection-related deaths and respiratory failure. The adenosine pathway component CD39 is undergoing evaluation in a phase II trial for neoadjuvant and adjuvant treatment with IPH520 (anti-CD39) combined with durvalumab and chemotherapy in treatment-naïve, resectable, early-stage NSCLC (NCT05742607). Additionally, uliledlimab, a CD73-inhibiting antibody, in combination with atelizumab, has entered Phase II trials (NCT05001347) after demonstrating clinical activity in both PD-L1 treatment-naïve and refractory cancer patients with high archival tumor expression of CD73.

While the growing roster of ICIs presents promise in cancer immunotherapy, a new concern arises in the form of immune-related adverse events (irAEs) associated with these treatments. irAEs can affect various organs, with manifestations ranging from rashes and diarrhea to pneumonitis, colitis, or hepatitis, and they can occur at any point during the treatment course. Due to a lack of understanding of the underlying causes of irAEs, glucocorticoid-mediated treatment is commonly employed for mitigation. Furthermore, the association of irAEs with clinical outcomes remains poorly elucidated. Some studies suggest a strong irAE is linked to a favorable outcome, indicative of a robust anti-tumor response [294], while others propose poorer outcomes among patients with early or specific irAEs [295]. Challenges persist in diagnosing certain irAEs like pneumonitis, and the optimal management of refractory irAEs remains unclear. More data are needed regarding managing irAEs in patients with autoimmune conditions, with some studies indicating that patients with a prior history of autoimmune diseases are not more likely to experience irAEs than other patients [296]. Management of irAEs now encompasses diverse approaches, such as utilizing anti-TNFα antibodies like infliximab or modulating the gut microbiome through fecal microbiota transplantation (FMT). The composition of the gut microbiome significantly influences the efficacy of anti-PD-1 therapy in individuals with PD-1-refractory melanoma. A clinical trial has unveiled a breakthrough in overcoming resistance to anti-PD-1 therapy among patients with PD-1-refractory melanoma. By integrating responder-derived FMT with anti-PD-1 therapy, researchers noted a significant improvement. Responders exhibited elevated levels of key taxa associated with anti-PD-1 response, along with heightened CD8^+^ T-cell activation and reduced frequency of interleukin-8-expressing myeloid cells, indicating a promising strategy for melanoma patients resistant to conventional anti-PD-1 therapy [297]. FMT has also demonstrated success in treating ICI colitis in select cases and is presently under investigation in a Phase I clinical trial (NCT04038619) [298]. Given the profound association of IL-6 with Th17 cells, recognized as significant contributors to inflammation in colitis or IBD, recent endeavors have sought to incorporate IL-6 blockade alongside dual PD-1 and CTLA-4 blockade to mitigate the irAE and enhance tumor immunity [299]. Presently, a clinical trial is underway to assess the efficacy of tocilizumab (anti-IL-6) in combination with nivolumab and ipilimumab (NCT04940299). A deeper comprehension of the relationships between irAEs and treatment outcomes, the identification of specific biomarkers for irAE diagnosis, the impact of tailored immunosuppression on ICI outcomes, and the management of steroid-refractory irAEs is imperative [300].

#### 3.1.2. Antibody–Drug Conjugates (ADCs)

ADCs are engineered to deliver a cytotoxic therapeutic payload, which, when used independently, can be highly toxic. However, in the case of ADCs, the payload is linked to an antibody that targets tumor cells. Once the antibody recognizes its target, the ADC is internalized via endocytosis and ultimately fuses with the lysosome. Here, the linker is enzymatically digested, releasing the cytotoxic drug into the cell to induce target cell death. To design an effective ADC, specific antigenic targets such as HER2, CD19, and CD33 are selected to precisely target tumor cells. The selection of the appropriate antibody depends on factors such as facilitating internalization, high antigen affinity, prolonged plasma half-life, and low immunogenicity [301]. To mitigate immunogenicity-related toxicity associated with murine antibodies, chimeric and humanized antibodies have replaced them [302]. The stability of ADCs is influenced by the type of linker used, which can be chemically labile, enzymatically labile, or non-cleavable [303]. Enzymatically labile linkers ensure specific drug release by being digested by tumor-specific enzymes, enhancing target specificity [304]. Non-cleavable linkers undergo proteolytic metabolism in the lysosome, releasing metabolites including the payload, linker, and amino acid appendages. They offer increased stability compared to cleavable linkers, with trastuzumab utilizing non-cleavable linker technology [305]. Payloads of ADCs typically consist of chemotherapeutic agents, such as topoisomerase inhibitors and derivatives of irinotecan, used in ADCs like Fam-trastuzumab deruxtecan, and sacituzumab govitecan, respectively. Ongoing developments aim to incorporate immunomodulatory agents as payloads to enhance the therapeutic window [306].

Currently, there are 15 FDA-approved ADCs on the market as of October 2023. Notably, T-DM1, composed of trastuzumab (anti-HER2) and the cytotoxic payload maytansine (DM1), was the first FDA-approved ADC for solid tumors [307]. Enfortumab vedotin (EV) targets nectin-4 and is approved for urothelial carcinoma, while sacituzumab govitecan is approved for metastatic TNBC, metastatic urothelial carcinoma (UC), and recently for hormone receptor-positive (HR^+^)/HER2-negative metastatic breast cancer [308]. Challenges in ADC therapy include the high toxicity of some therapeutic agents and the development of resistance mechanisms, such as target downregulation and defects in internalization and trafficking of ADCs, as well as mutations affecting payload sensitivity and antigen recognition [302]. This leads to the utilization of ICIs in combination with ADCs to enhance response, along with mAb-mediated blockade of target antigens [309]. Moreover, ongoing developments include the creation of bispecific ADCs targeting two distinct antigens. A recent focus has been on Claudin 18.2 as a new target for ADC development in individuals with solid tumors expressing this antigen. RC118, designed to target solid tumors in patients with positive Claudin 18.2 expression, received orphan drug designation from the FDA in 2022 [310].

#### 3.1.3. Bispecific Antibodies (bsAb)

Originating from the concept of Nisonoff and colleagues in the 1960s, bispecific antibodies have evolved into effective tools by recognizing two distinct epitopes [311]. Functionally bsAbs have been categorized as combinatorial bsAbs, where the function of the bsAb is a combination of the individual functions of the two parent antibodies and obligate bsAbs, in which the bsAb acquires a novel activity that is not displayed by any of the parent antibodies [312]. The structural journey of bsAbs primarily addresses therapeutic effectiveness. Designing the most functionally effective bsAb from various combinations of two light (V_L_) and two heavy chains (V_H_) poses a significant challenge known as the “chain association issue”. Architectural modifications to tackle this challenge can result in differences in valence among bsAbs ranging from bivalent to tetravalent, with a maximum of trivalent for each target. Based on architectural format, the bsAbs can be classified into three classes. In the most minimalistic approach, only the single-chain variable fragments (Fv) are combined in the absence of the constant fragment (Fc) [313]. Although this format has better tissue penetrability due to low molecular weight, they are less stable, with a short half-plasma life, and unable to perform antibody-dependent cell cytotoxicity (ADCC) due to the lack of Fc. In the second generation with a symmetric format or appended IgG/IgG-like format, antigen-specific moieties are combined in a single polypeptide chain or one HL pair, keeping the Fc region intact [314]. They are mostly tetravalent, and due to their large size, they have low tissue penetrability. The third is the asymmetric format or the preserved IgG native format, with an intact Fc domain and extended plasma-life.

Based on the target, bsAbs can be classified into three primary types: those designed to target two different tumor antigens, those aimed at targeting one tumor antigen along with one immune-related molecule, and those intended to target two distinct immune-related molecules. Bispecific T-cell engagers (bsTCE) mainly belong to the second category [312]. In the current landscape, most of the ongoing trials focus on bsTCEs, which will be the specific focus of this review. The primary function of bsTCEs is to simultaneously engage tumor cells and T cells, leading to MHC-peptide-independent activation of T cells. These antibodies exhibit specificity towards a variety of molecules, encompassing immune inhibitory targets like PD-1, CTLA-4, CD73 [315], immune-stimulatory molecules such as OX-40, CD28, and tumor-associated proteins like melanoma-associated antigen (gp100) [316], epithelial cell adhesion molecule (EpCAM) found on tumor cells, CD33 expressed on MDSCs, or Delta-like ligand 3 (DLL3) [317] to facilitate direct lysis of tumor cells or immunosuppressive cells. Most FDA-approved bsTCEs typically incorporate a CD3-targeting domain to stimulate T-cell signaling enabling the discharge of cytotoxic agents, effectively overcoming the issue of diminished tumor antigen presentation caused by MHC downregulation on tumor cells. Compared to alternative bsAb formats, bsTCEs demonstrate higher efficacy in tumor cell lysis at a lower T-cell-to-target ratio in vitro [312]. Initial clinical testing of bispecific T-cell engagers showed nonspecific cytotoxicity, halting research until the first clinical trial data on blinatumomab demonstrated efficacy in treating acute lymphoblastic leukemia [318]. This paved the way for FDA approval of blinatumomab in 2014 for relapsed/refractory B-cell ALL and in 2023 for minimal residual disease-positive B-cell ALL [319]. Currently, there are approximately seven FDA-approved bsTCEs for cancer immunotherapy.

While bsTCEs targeting T cells and TAAs show effectiveness in hematologic malignancies, their performance in solid tumors poses challenges. The engagers remain constitutively active within the TME. The use of probody technology, which involves masking the binding site until cleaved by tumor proteases, enhances specificity, resulting in lower toxicity levels [320]. Counteracting TME-mediated immune suppression can be achieved with dual IC-specific bsAbs such as volrustomig, which targets PD-1 and CTLA-4. Studies have demonstrated its efficacy and safety profile comparable to dual ICB therapy in renal cell carcinoma (RCC), and it is currently undergoing investigation in multiple trials (NCT05775159, NCT06079671) (Table 1). Preclinical studies have demonstrated that targeting multiple proteins, such as LILRB1, LILRB2, KIR3DL1, and CD47 receptors, uniquely engages myeloid cells positive for LILRB1/2. This approach guides immune cells to tumor sites by targeting CD47 checkpoint receptors expressed on cancer cells. The first-in-class inhibitor, which targets all three receptors, is being investigated in clinical trials (NCT05788484, NCT05763004). DLL3, an inhibitory notch ligand expressed in most neuroendocrine cancers (NECs), including SCLC, has emerged as a promising target for bsTCE [317] and is undergoing evaluation in a Phase I study for SCLC and NEC (NCT04429087) [321]. BsAbs, like other antibody-based therapies, can induce severe immune-related cytotoxicities such as CRS and ICANS, arising from T-cell hyperactivation and cytokine release [322]. To mitigate toxicity while enhancing anti-tumor efficacy, strategies include using engagers with low CD3 affinity to decrease T-cell activation, as demonstrated by an anti-CLL1 bispecific with one low-affinity CD3 arm [323]. Using costimulatory molecules like CD28 or CD137 alongside a tumor antigen instead of CD3, specifically targeting a subset of antigen-experienced T cells, reduces toxicity [324].

Other cytotoxic immune cells like γδ T cells and NK cells are being investigated for bispecific targeting. The Vδ2 subset of γδ T cells, known for their pro-inflammatory and anti-tumor properties, are particularly of interest. Several γδ T cell-engaging molecules, such as gammabodies, have been developed to activate Vγ9Vδ2 T cells against tumors, with one undergoing a Phase I/IIa trial (NCT05369000) for therapy-refractory metastatic castration-resistant prostate cancer (mCRPC). Similarly, in NK cells, efforts have been directed towards developing bispecific or trispecific NK cell engagers (TriKE) targeting NKG2D, NKp46, NKp30 with or without CD16 targeting, and tumor antigens like CD30 and CD33 [325]. Therefore, due to its significant therapeutic potential and its role as an excellent off-the-shelf tool for unleashing the anti-tumor potential of immune cells, bispecific engagers remain an excellent target for the future.

#### 3.1.4. Other Monoclonal Antibody (mAb)-Based Therapies

mAb-based therapies serve as direct immune-mediated killers of tumor cells or as agonists to stimulate costimulatory receptors, enhancing T cell-mediated cytotoxicity [280]. Several FDA-approved mAbs employ mechanisms such as antibody-dependent cellular cytotoxicity (ADCC), complement-dependent cytotoxicity (CDC), and antibody-dependent cellular phagocytosis (ADCP) to target a range of antigens, including CD20 (rituximab, ofatumumab, obinutuzumab), CD38 (daratumumab, isatuximab), CD52 (alemtuzumab), SLAMF7 (elotuzumab), HER2 (trastuzumab, pertuzumab), EGFR (cetuximab, panitumumab, necitumumab), GD2 (dinutuximab), and CCR4 (mogamulizumab), for treating hematological and solid malignancies [326]. Notably, rituximab was the first FDA-approved mAb for the treatment of follicular lymphoma, diffuse large B-cell lymphoma, and chronic lymphocytic leukemia. Induction of apoptosis by blocking receptor dimerization to prevent growth factor receptor signaling is also used as a mechanism of targeted cell killing. Cetuximab prevents EGFR homo-dimerization/signaling, in contrast to trastuzumab which prevents hetero-dimerization of HER2 with other growth factors, leading to apoptosis of tumor cells in ovarian and breast malignancies [327,328,329]. CD38-targeted antibodies for multiple myeloma induce apoptosis, ADCC, and phagocytosis while depleting CD38^+^ immune regulatory cells [330]. IgG1 mAbs primarily interact with activating Fc gamma receptors such as CD16A on NK cells to facilitate ADCC. While NK cells serve as the primary effectors of ADCC, macrophages, neutrophils, and other myeloid cells can also contribute to this process. The CD52 antibody alemtuzumab induces complement-dependent cellular lysis and has shown clinical significance in conditions such as chronic lymphoblastic leukemia (CLL), cutaneous T-cell lymphoma, peripheral T-cell lymphoma, and T-cell prolymphocytic leukemia. mAbs targeting pro-angiogenic factors, such as bevacizumab against VEGF and amucirumab against VEGFR2, are utilized either alone or in combination with other therapies for the treatment of various malignancies, including colorectal cancer, recurrent glioblastoma, metastatic hepatocellular carcinoma, non-squamous NSCLC, and renal cell carcinoma [331,332]. Another immunosuppressive cytokine, the IL-8 pathway, is involved in the infiltration of neutrophils and PMN-MDSC in the TME [333], and a high serum level is associated with poor prognosis in pancreatic adenocarcinoma [334] and resistance to combination therapy with ipilimumab and nivolumab in patients with advanced cancer [335]. Several antibodies targeting IL-8 and the IL8 receptor (CXCR2) are currently under investigation in different ongoing trials, in combinations with other immunotherapeutic agents (NCT02499328, NCT04050462, NCT03689699).

Another class of mAb-based immunotherapeutic agents are radioimmunoconjugates (RICs), where mAbs are linked to radionuclides for cancer therapy and imaging. Unlike ADCs, RICs do not rely on cellular uptake; instead, the radionuclide emits radiation upon binding to tumor cells, inducing DNA damage. These specific combinations of radioneucleotides with antibodies allow specific targeting and efficient killing of tumor cells. Currently, two FDA-approved RICs are ibritumomab tiuxetan, labeled with yttrium-90 (90Y), and 131I-tositumomab, labeled with iodine-131, which target CD20^+^ B cells in non-Hodgkin’s lymphoma (NHL) [336,337]. 177Lu-PSMA-617 has shown promising results in Phase III clinical trials for prostate-specific membrane antigen-positive metastatic castration-resistant prostate cancer and received FDA approval in 2022.

Several antibodies targeting costimulatory molecules on immune cells have been trialed to promote tumor-specific T-cell immunity. Agonistic antibodies against costimulatory molecules have been tested in various formats, including bsAbs or combination therapies. Clinical trials of INCAGN01949, a fully human anti-OX40 agonistic mAb, in ovarian, NSCLC, and colorectal cancer, demonstrated no observed toxicity. However, these trials showed limited T-cell infiltration in tumors and no increase in circulating effector T cells when used as monotherapy [338]. Phase I data from a combination approach involving CD137/4-1BB and OX-40 agonists and ivuxolimab in NSCLC and melanoma (NCT02315066) demonstrated good tolerability and preliminary anti-tumor activity in specific patient groups [339]. In glioblastoma, the investigation of combination therapy involving ICIs with CD137 agonistic antibodies is underway (NCT02658981). Bintrafusp alfa, a first-in-class bifunctional fusion protein, demonstrated promising clinical efficacy and a well-tolerated safety profile in pre-treated NSCLC patients with anti-PD-L1 antibodies, achieved by PD-L1 blockade and TGF-β entrapment [340]. Multiple ongoing trials are assessing the efficacy of this agent across various solid tumors (see Table 1).

### 3.2. Adoptive Cell Transfer (ACT) Therapy

Currently, there are several CAR-T therapies that have received approval, with numerous biotech and pharmaceutical companies actively engaged in adoptive cell transfer trials.

#### 3.2.1. Tumor-Infiltrating Lymphocyte (TIL) Therapy

TIL therapy harnesses tumor-reactive lymphocytes from patients through adoptive cell transfer. However, due to the limited availability and expansion potential of tumor antigen-specific cytotoxic T cells in the immunosuppressive TME, they may not suffice for tumor eradication. The advantage of TIL therapy lies in isolating tumor antigen-reactive cytotoxic T-cell populations from resected tumors, expanding them in vitro, and reintroducing them into the patient to initiate anti-tumor responses. This approach broadens the pool of tumor-reactive cells, as the TIL population comprises T cells reactive against multiple tumor antigens, including neoantigens and tumor-associated antigens (TAAs) [341]. On 16 February 2024, the FDA approved Lifileucel as the first TIL therapy for solid tumors, offering a treatment option for advanced melanoma following anti-PD-1 and targeted therapy. Another TIL therapy product, LN-145, in a Phase II trial (NCT03108495), successfully treated patients with advanced cervical cancer and has received breakthrough therapy designation for this indication. Another Phase II trial is evaluating IL-2-stimulated TIL infusion in metastatic pancreatic, ovarian, and colorectal cancer (NCT01174121) (Table 2).

Several challenges exist in the expansion and administration of TILs. In the immunosuppressive TME, the identification of tumor antigen-reactive cells can be challenging due to potential exhaustion or dysfunction, leading to limited cells available for expansion. Various studies have identified markers such as CD103, CD39, and PD-1 on CD8 T cells as indicative of tumor reactivity [342,343]. However, it is important to note that PD-1 and CD39 are also associated with T-cell exhaustion and dysfunction. Other markers, such as the costimulatory CD137/4-1BB, are being considered based on their expression on tumor-reactive CD8 T cells [344].

Prolonged expansion of isolated TILs in vitro can lead to loss of stemness, exhaustion, low proliferation, and shorter telomeres, indicating a reduced overall lifespan. To address these shorter cultures for expansion of unselected TILs in a bulk population of different lymphocytes appears to be beneficial compared to selective expansion methods [345,346]. Combination with IL-2 has shown improvement in therapeutic outcomes, but the conflicting role of IL-2 in driving Treg differentiation and T-cell dysfunction, along with systemic toxicity, remains a challenge [347]. Ongoing studies are investigating the modulation of IL-2 dosage to minimize toxicities without compromising the anti-tumor effect [348]. Additionally, including PD-1/CTLA-4 inhibitor therapy before TIL therapy has shown benefits by increasing the number of tumor-reactive TILs post-ICI treatment [349]. In conclusion, while TIL therapy holds promise as a personalized immunotherapeutic approach for solid tumors, addressing challenges in TIL expansion, identification of tumor-reactive cells, and managing associated toxicities remains critical for optimizing its clinical efficacy and broadening its applicability in cancer treatment.

#### 3.2.2. Chimeric Antigen Receptor (CAR)-Based Therapy

##### CAR-T

One of the trailblazers in adoptive cell transfer therapy is CAR-T cells, pioneered by the team led by Dr. Carl June starting the therapeutic journey with FDA approval in 2017. Since then, the CAR-T platform has evolved through four generations, primarily focusing on advancements in the intracellular domain, with the fifth generation currently under development. The first generation of CAR-T cells comprised an extracellular single-chain variable fragment and an intracellular CD3€ chain but lacked costimulatory signaling and hence showed low proliferation, diminished cytokine production, and reduced lifespan, necessitating IL-2 supplementation for cell proliferation and persistence [350]. In the second-generation CARs, the addition of a costimulatory domain with 4-1BB or CD28 enhanced cell persistence and improved anti-tumor responses and increased remission rates in leukemia [351]. However, with 4-1BB domains causing early exhaustion and CD28 in CARs inducing a constitutively active state, third-generation CARs were developed featuring multiple costimulatory domains like CD28/4-1BB or CD28/OX-40 [352]. While these CARs exhibited better persistence and proliferation, failure to demonstrate improvement in hematologic malignancies prompted the targeting of next-generation CAR-Ts towards solid tumors [353]. Currently, FDA-approved CAR-Ts for non-Hodgkin’s lymphoma, B-cell acute lymphoblastic leukemia, and multiple myeloma are based on second-generation CAR-T cells. Fourth-generation CAR-T cells incorporate an NFAT-responsive cassette to express transgenic cytokines upon target engagement, enabling TRUCK (T-cell redirected for universal cytokine-mediated killing) CAR-T cells to produce inflammatory cytokines that enhance T-cell penetration and overcome immunosuppression in the TME [354]. The ongoing development of fifth-generation CARs is focused on addressing off-target effects and toxicity. Current approaches include adding IL-2 receptors to enable antigen-dependent JAK/STAT pathway activation and switch receptors, where an ON-switch drug activates CAR-T cells and an OFF-switch leads to CAR-T depletion [355]. Three types of tumor recognition circuits for simultaneous recognition of multiple antigens, the AND-gate circuit, NOT-gate circuit, and OR-gate circuit, have been proposed [356]. The AND-gate circuit activates CAR-T cells using a combination of antigen-recognizing receptors, ensuring targeted killing; however, it may spare tumor cells expressing only one antigen [357]. For the NOT-gate strategy, a combination of a CAR (for tumor cell killing) and an inhibitory CAR on the same cell is used. This combination targets non-specific cells through the inhibitory CAR, while specific tumor targets are identified by the presence of the CAR and the absence of the inhibitory CAR [358]. The OR-gate strategy employs two different antigen recognition domains to recognize the tumor by at least one of the antigens.

In B-cell lymphoma, approximately 20–25%, and in B-ALL, about 6–68% of disease relapses are attributed to antigen-mediated immune escape [359]. This is characterized by low tumor antigen (CD19) expression on target cells, which reduces anti-tumor effectiveness, hampers disease remission, and is known as antigen-negative relapse [360]. In cases of antigen-positive relapse, CAR-T efficiency is limited in certain tumors characterized by low tumor persistence or low disease remission. Strategies to tackle these challenges include dual antigen targeting, such as CD19/CD20, and the utilization of bsAbs. These approaches can be applied either in conjunction with CAR-T cells or in designing bispecific TCR-CAR-T therapies [361]. While the CD19/CD20 approach has shown promise in treating B-cell malignancies, efforts targeting CD19/CD22 have not yielded comparable success [362]. Currently, the antigen-binding domain of CARs are derived from mouse antibodies, raising the possibility of an anti-CAR-T immune response post-infusion. The development of fully human CAR-T cells has shown some improvement in their persistence, but they are yet to be translated into clinical trials [363]. An alternative approach entails substituting the single-chain variable fragment (scFv) with a heavy chain. Although not prospectively compared, patients treated with this product have shown higher overall response rates and longer response durations compared to those who received the scFv-containing CAR [364].

While a significant number of FDA-approved CAR-Ts target hematological malignancies, ongoing trials are targeting solid tumors (Table 2). Ongoing studies focus on engineering CAR-T cells to improve solid tumor penetration by equipping them with ECM-degrading proteins [365], enhancing adhesion molecule expression, administering vasculature-disrupting agents, and arming CAR-T cells with IL15 and IL21 [366]. Engineered CXCR1/CXCR2-expressing CAR-Ts are showing promise in preclinical models [367]. Another challenge is the selection of the right candidate as a target antigen. CLDN6 specific CAR-T cells also showed efficacy in a Phase 1/2b trial when used in combination with an mRNA vaccine [368]. Other tumor-associated antigens identified in pediatric brain tumors (PBTs), including B7-H3, EGFR, Herceptin-2 (HER2), disialoganglioside 2 (GD2), are in clinical testing. Currently, NKG2D is emerging as an attractive target for solid tumors. Unlike other checkpoints, NKG2D activates T cell and NK cell-mediated cytotoxicity. Since most tumors upregulate NKG2D ligands such as MICA/MICB or ULBP1-6, NKG2D CAR-T cells can effectively target tumor cells utilizing these NKG2D ligands [369]. Apart from that, to overcome the inhibitory effects of the TME, using ICI for blocking PD-1 and administering adenosine A2BR agonist BAY 60-6583 with CAR-T has demonstrated efficacy in preclinical and clinical studies by preventing exhaustion and improving anti-tumor effect [370]. Additionally, engineering CAR-T cells to release pro-inflammatory cytokines like IL15, IFN-γ, IL-18, and IL-12 or integrating the CD28 domain in the CAR structure to stimulate the production of inflammatory cytokines has proven to be beneficial [371].

CAR-T cell therapy faces toxicity challenges like cytokine release syndrome (CRS) and immune effector cell-associated neurotoxicity (ICANS), which can be managed with drugs like tocilizumab or engineered safety switches. Tocilizumab is effective against CAR-T cell-mediated CRS in B-ALL and has been FDA-approved for the treatment of life-threatening CRS induced by CAR-T cells [372]. Inducible suicide genes like Fas or iCaspase-9 engineered into CAR-T cells and activated by a chemical inducer of dimerization (CID) to trigger apoptosis of the infused CAR-T cells provide an emergency safety switch to control toxicity [373]. Manufacturing optimizations like using less differentiated, central memory, or stem-like memory CAR-T cells, which have improved durability, by modulating culture conditions with cytokines or factors like inosine to maintain stemness can enhance CAR-T cell effectiveness due to their better durability in vivo [374]. Another remaining challenge is the absence of readily available off-the-shelf CAR-T cells for therapy, primarily due to the MHC restrictions of T cells. Currently, only autologous CAR-T cells are utilized for therapeutic applications to mitigate the risk of graft-versus-host disease (GVHD) associated with allogeneic CAR-T cells. This obstacle can potentially be addressed by genetically deleting the T cell receptor (TCR) genes and MHC I and MHC II-associated genes from allogeneic CAR-T cells. Furthermore, equipping these cells with HLA-E expression can offer protection against immune attacks from the host. However, the absence of self-MHCs on allogeneic CAR-T cells may render them susceptible to targeting by the host’s NK cells [375].

In November 2023, concerns emerged regarding T-cell malignancies in patients treated with autologous CAR-T cells. The FDA launched an investigation into 20 reported cases among 34,400 patients who received CAR-T cell treatment. However, there is limited data on the patients’ immune status, including previous immunotherapy history, clonal hematopoiesis instances, age, and existing immunosuppression. Previous occurrences of clonal hematopoiesis may increase the risk of prolonged cytopenia after CAR-T cell therapy. Current investigations indicate that reported cases of T-cell malignancies from CAR-T cell treatment occur at a lower rate compared to secondary cancers seen with conventional chemotherapy, indicating the benefits outweighing the risk of approved CAR-T therapies [376]. However, further research is necessary to understand the mechanisms underlying the risk of malignancies and to develop preventive measures. Alternative CAR-based therapeutic approaches modified from NK cells and γδT cells are also being tested. Overall, a lower incidence of CRS cases has been noted in the case of CAR-NK cells compared to CAR-T cells [377]. Interestingly, this opens the avenue for another class of CAR cells.

##### CAR-NK

As discussed in the previous section on CAR-T cells, there are emerging engineered cell platforms that explore non-MHC-based recognition while addressing problems associated with engineered T cells. Two such platforms are CAR-NK and CAR- γδ cells [378]. Unlike T cells, the anti-tumor activity of NK cells relies on a system of activating and inhibitory NK cell receptors (aNKR and aiNKR, respectively) instead of MHC-based peptide recognition. Killer immunoglobulin-like receptors (KIRs) recognize self-MHC to maintain tolerance, but tumor cells can downregulate MHC, creating a “missing self” and reducing inhibitory signals. This allows activating signals from NKG2D and NKp46 binding NKG2DL-like ligands to prevail, triggering NK cell cytotoxicity against tumor cells [378]. NK cells inherently possess lytic granules, necessitating an optimal spatiotemporal mechanism for cytotoxic activation. Hence, NK cells exhibit natural cytotoxicity against a broader range of targets and are less susceptible to tumor escape mechanisms compared to CAR-T cells [379]. Interestingly, a lower incidence of CRS has been observed in CAR-NK treatment, which could be partially attributed to the diverse cytokine profile of NK cells. All of these factors made NK cells an attractive target for CAR-NK generation and most importantly for engineering off-the-shelf allogenic CAR-NKs [379]. Like CAR-T cells, CAR-NK cells face challenges with persistence and tumor infiltration in solid tumors, but engineering CAR-NKs to secrete cytokines like IL-15 or stimulation with IL-12/15/18 improves persistence [380,381]. Modifying CAR-NKs with an NFκB-inducible IL-12 promoter (TRUCK) or expression of CXCR1 also enhances tumor homing, cytotoxicity, and monocyte recruitment by the CAR-NK [382]. Replacing the CAR scFv with NKG2D or nanobodies further augments the potency of CAR-NKs against solid tumors [369]. Allogenic CAR-NK cells are in trial with some showing manageable safety profiles, and preliminary anti-tumor activity and disease target encompasses hematologic malignancies as well as solid tumors. Some studies utilize high-affinity NK (haNK) cell lines for allogeneic CAR-NKs possessing a high-affinity CD16/FcγRIIIa (158V) allele and IL-2 retained in the endoplasmic reticulum (ER) [383]. These cells demonstrate potent cytotoxicity owing to their abundant granulysin and perforin reserves.

Understanding NK exhaustion profiles will aid in determining combinatorial therapeutic approaches to improve CAR-NK function and persistence and, like CAR-T cells, standardized protocols for CAR-NK cell manufacturing, lymphodepletion, and administration schedules need to be established for clinical application. Currently, about 30 different clinical trials are ongoing to test the efficacy of CAR-NK cells in solid and hematologic malignancies (Table 2). With the potential advantages of NK cells over T cells as targets for CAR-based therapies, addressing current challenges is crucial for developing CAR-NKs as another tool in the immunotherapeutic repertoire.

#### 3.2.3. T-Cell Receptor-Engineered T Cells (TCR-Ts)

While CAR-T cells represent a notable advancement, their efficacy has predominantly been showcased in hematologic malignancies, with persistent challenges in addressing solid tumors. TCR-T technology emerges as a promising alternative, relying on engineered TCRs to recognize MHC-bound peptides. This recognition triggers signaling cascades, resulting in T-cell activation, cytokine secretion, and cytolytic activity against the target. The HLA-restricted recognition of antigens provides TCR-T with the advantage of targeting proteins from various cellular compartments, including neoantigens, tumor-associated antigens (TAAs), and viral oncoproteins [356,384]. The signaling synapse with T-cell coreceptors and TCRs leads to the recruitment of proximal signaling kinases, enhancing signaling kinetics in TCR-T cells. This results in remarkable signaling sensitivity and receptor capacity for the TCR-T [385]. Hence, only a small number of antigen molecules can trigger an effective signaling cascade.

In TCR-T development, antigen selection prioritizes tumor-exclusive targets with validated HLA-restricted epitopes to minimize off-tumor cytotoxicity while optimizing TCR affinity to prevent early exhaustion [386]. During TCR assembly, strategies like substituting human constant regions with murine counterparts, using CRISPR-Cas9 to replace endogenous chains, or genetically knocking out endogenous chains mitigate mispairing, along with enhancing stability and homogenizing expression through disulfide bond addition or hydrophobic sequence incorporation [387,388]. Subsequently, transgenic TCR-expressing T cells are expanded ex vivo with cytokines to promote stemness and cytotoxicity, producing an optimized TCR-T-cell population for adoptive cell therapy [389]. Among the shared challenges linked to other engineered T-cell platforms, off-tumor toxicities can occur when the target is a TAA like MART-1 or gp100 in melanoma, where all melanocytes express these two proteins. Another concern is cross-reactivity to epitopes derived from off-target proteins. For instance, epitopes derived from MAGE-A12 and TITIN are also recognized by TCRs designed for the recognition of MAGE-A3 epitopes, leading to severe cardiotoxicities [390,391]. Additionally, a crucial factor for the success of TCR-T therapy is the consistent expression of MHCI proteins for effective antigen presentation to the cytotoxic TCR-T cells. However, many tumors downregulate HLA genes or genes associated with the antigen presentation process, like TAP1, TAP2, IFNγR, or show loss of HLA heterozygosity in tumor cells, affecting the antigen-presenting machinery [384,392]. Like CAR-T, an essential prerequisite for successful ACT treatment is the trafficking of the infused cells to the tumor, which is regulated by chemotactic signaling [356]. Increased efficacy in tumor infiltration of IL-8 receptor CXCR2-expressing MAGE-A3-specific TCR-T was demonstrated in preclinical models [393]. Currently, over 100 trials are ongoing in different malignancies, with about 31 in solid tumors (Table 2). Mesothelin-targeted TCR-T cells have shown manageable toxicity profiles and high rates of disease control in advanced mesothelin-expressing cancer patients (NCT03907852) (Table 2) [394]. Another KRAS^G12V^-specific TCR-T with a tolerable safety profile is currently being evaluated in KRAS^G12V^-expressing solid tumors (NCT06105021) (Table 2). However, further research is required to enhance the tumor-infiltrating properties of TCR-T cells. Additionally, challenges associated with product manufacturing and pre-infusion procedures like lymphodepletion also require fine-tuning to maximize the efficacy of the final product.

### 3.3. Cancer Vaccines

The traditional notion of vaccines primarily targeting non-self pathogenic antigens has shifted, with cancer vaccines now emerging as a promising therapeutic avenue. Cancer vaccines operate on the principle of antigen recognition and presentation by the body’s own APCs after immunization, leading to the activation of T cells capable of combating malignant cells upon reaching the tumor site [395]. While many cancer vaccines remain in preclinical or clinical research stages, only two preventive and two therapeutic cancer vaccines have gained FDA approval. However, data from ongoing trials indicate promising developments on the horizon for the field. The categorization of cancer vaccines involves three primary aspects: (i) types of antigen targets, (ii) platforms utilized for immunization, and (iii) applications that involve prophylactic or therapeutic vaccines.

Antigen selection for vaccination considers several factors, such as high immunogenicity to evoke a robust and persistent response from CD4 helper and CD8 cytotoxic T cells, as well as tumor-specific or tumor-exclusive expression of the antigen, to ensure effective targeting and minimize off-target toxicity [396]. The antigen targets for cancer therapeutics encompass tumor-associated antigens (TAAs) and tumor-specific antigens (TSAs). TAAs typically consist of non-mutated self-antigens with elevated expression levels in tumors compared to healthy tissue, such as melanoma-associated antigen (MAGE) [397]. TSAs, on the other hand, can be self-tumor antigens like epidermal growth factor receptor variant III (EGFRvIII), expressed in 25% of EGFR-expressing glioblastoma [398], or non-self viral antigens like human papillomavirus E6 and E7 proteins (HPV E6 and E7), expressed in approximately 60% of oropharyngeal cancers and almost all cervical cancers [399]. Both TSAs and TAAs can constitute a group of antigens called shared antigens, which need to be expressed in a sufficient proportion of patient groups of a certain cancer type to act as antigen targets for prophylactic or therapeutic vaccine design. EGFRvIII, which is expressed in 25% of EGFR-expressing glioblastomas, or the E6 and E7 proteins of HPV, which are expressed in approximately 60% of oropharyngeal cancers and almost all cervical cancers, or MAGE are all targets for shared antigens [398,399].

A newly defined group of TSAs, known as neoantigens, has emerged as promising targets for personalized cancer vaccines (PCVs) due to their robust expression in tumors and high immunogenicity. Neoantigens are non-germline coded proteins that can arise due to non-synonymous somatic mutations of coding regions, human endogenous retroviruses, frameshifts occurring in microsatellite-instability-high tumors, or post-translational modifications [40,400,401]. Due to their non-germline origin, they can avoid inducing T-cell central tolerance [400,402]. As most of them are highly immunogenic, the likelihood of inducing peripheral tolerance while priming naïve peripheral T cells is low, making them attractive candidates for PCVs [401]. Nevertheless, in determining the efficacy of a PCV, accurately predicting the relevant neoantigens becomes paramount to elicit an optimal anti-tumor response and ascertain whether any of these neoantigens possess the ability to induce peripheral tolerance. One study reveals the presence of intratumorally expanded Treg TCRs exhibiting specificity towards neoantigens [403]. Although the mechanisms of formation of the Tregs remained unknown in the case of neoantigen-specific Tregs, it is important to understand if a section of neoantigens can induce immune tolerance peripherally.

Vaccines can be categorized based on their delivery platform, which includes whole-cell-based, nucleic acid-based, peptide-based, and virus-based vaccines. Here is a brief overview of each category:

#### 3.3.1. Cell-Based Cancer Vaccines

The cell-based platform involves transferring antigens via autologous DCs pulsed with the antigen by mRNA electroporation, lentiviral transfection, fusion with tumor cells, or incubating with the whole lysate of the tumor. The Phase III clinical trial of DCVax-L, an autologous tumor lysate-incubated DC vaccine, showed significantly higher OS in glioblastoma groups [404]. Currently, the only FDA-approved therapeutic vaccine is sipuleucel-T, for the treatment of prostate cancer [405]. To improve the efficacy of this platform, it is crucial to refine the generation, maturation, activation, and lymphoid-homing capability of DC subsets. Additionally, combining this approach with checkpoint inhibitors or T cell therapy has the potential to further augment its efficacy [396]. Careful antigen peptide selection and inclusion of both MHC class I and II epitopes can elicit coordinated CD4^+^ and CD8^+^ T-cell responses for optimal anti-tumor immunity.

#### 3.3.2. Peptide-Based Cancer Vaccines

Another platform is peptide-based delivery, which involves antigen-derived amino acid sequences of 8–10 peptide length containing a MHCI binding short peptide. Additionally, synthetic long peptide (SLP) vaccines consist of approximately 25–35 amino acids, encompassing multiple epitopes, thereby increasing stability and the number of epitopes to elicit a broader response. Peptide-based cancer vaccines provide customizable and safe immunotherapy options as they can be precisely tailored with minimal epitopes to elicit targeted immune responses. These vaccines offer the advantage of synthetic manufacturing, ensuring complete identification and elimination of biological contaminants associated with traditional antigen sources. Peptide-based vaccination, while advantageous in some aspects, face challenges such as short half-life, inadequate immunogenicity, susceptibility to degradation, and limited bioavailability [406]. NeuVax™, the leading peptide-based breast cancer vaccine, reached Phase III clinical trials initiated by the US National Cancer Institute in 2011, marking a significant advancement in its development and evaluation [407]. Despite clinical studies on various SLPs for cancer, including combinations, no regimen has yet achieved an improved overall response rate (ORR), underlining persistent challenges in enhancing treatment effectiveness.

#### 3.3.3. Viral- and Bacterial-Vector-Driven Cancer Vaccines

Viral and bacterial vectors are also being explored as cancer vaccines due to their inherent immunogenicity. As a standalone vaccine, this platform shows limited efficacy in clinical trials; however, combination with other chemotherapeutic agents demonstrated improved outcomes. For instance, an engineered herpes virus encoding GM-CSF combined with chemotherapy achieved high response rates in breast cancer patients. Overall, viral and bacterial vector cancer vaccines have shown promise in preclinical studies and select clinical trials, particularly when used in combination regimens [408]. However, further optimization is necessary to improve clinical efficacy as monotherapies. Their intrinsic immunogenicity and ability to deeply penetrate the TME make them attractive delivery platforms if limitations can be overcome.

#### 3.3.4. Nucleic Acid-Based Cancer Vaccines

Nucleic acid-based vaccines encompass DNA and RNA vaccines. DNA vaccines are particularly appealing as they offer an easy and attractive target platform. They have the capability to simultaneously deliver multiple antigens, as seen in polyepitope DNA vaccines, and can incorporate built-in adjuvants such as CpG and other immune stimulatory cytokines or chemokines. Clinical studies have demonstrated the immunogenicity of DNA vaccines in the early phases of trials. However, DNA vaccines have achieved limited success overall in clinical trials for cancer due to their poor immunogenicity [409]. With recent advancements such as the Nobel Prize in Physiology and the breakthrough developments in COVID-19 vaccines and personalized cancer vaccines, mRNA vaccines have profoundly revolutionized vaccination platforms and therapeutic approaches toward cancer (Table 3). In the latest clinical trial of mRNA-4157, encoding up to 34 different neoantigens, in high-risk melanoma, the combination with mRNA-4157 and anti-PD-1 significantly prolonged distant-metastasis-free survival and reduced the risk of developing distant metastases or death by 65% compared to pembrolizumab alone [410]. Due to these promising clinical findings, mRNA-4157 has been granted breakthrough designation by the FDA for the treatment of resected III/IV melanoma for patients with a high risk of recurrence, making it the world’s first mRNA vaccine to enter Phase III trial study. In another Phase I trial conducted in surgically resected PDAC patients, sequential administration of PD-L1 immune checkpoint inhibitors, individualized mRNA vaccine encompassing 20 different neoantigens, and four chemotherapy regimens resulted in half of the vaccine recipients, all of whom had expanded neoantigen-specific T cells after vaccination, remaining cancer-free [411]. This approach generated cytotoxic and durable neoantigen-specific T cells, potentially delaying recurrence in patients with PDCA. For cancer with some of the highest post-resection recurrence and mortality rates and an immunosuppressive TME that has frustrated immune-targeted interventions, these findings are promising. The advantages of mRNA-based vaccines include ease in the development process. Exogenous mRNAs can be inherently immunostimulatory, acting as an adjuvant to drive DC maturation and eliciting T cell responses; however, innate immune sensing may negatively affect immune responses. Thus, modulating the immunogenicity of mRNA vaccine formats can be useful. Additionally, optimizing the delivery mechanisms of mRNA vaccines is crucial, as they must penetrate the lipid membrane barrier to reach the cytoplasm for translation to functional protein. Critical factors for optimizing mRNA vaccine efficacy include mRNA sequence modification to improve stability for effective storage, transport, and administration at ambient temperatures, as well as translation efficiency in cells and delivery methods like cationic liposomes or protamine complexes to protect mRNA and facilitate antigen-presenting cell uptake [412,413]. With the demonstrated efficacy of this platform for personalized cancer vaccines, further optimization of current conditions and addressing current challenges can potentially transform this into a powerful platform for PCVs.

Overall, with the current progress in personalized cancer vaccine approaches, the selection of the right vaccine delivery platform is still ongoing. Other platforms, such as neoantigens encoded within a chimpanzee adenoviral vector and DNA-based platforms are also under development for designing personalized cancer vaccines for solid tumors. Optimization of the vaccine format, including adjuvant selection, administration schedule, and administration route, needs further understanding. Additionally, to maximize efficacy, targeting patients at the minimal residual stage of the disease is crucial as it avoids the effects of strong tumor-based immunosuppression and allows for an unrestricted priming and anti-tumor response time. Furthermore, exploring the most effective combination approaches, such as ICIs with vaccines or adoptive T-cell therapy with neoantigen-specific expanded T cells post-vaccination, can be further examined to establish a more synergistic therapeutic regimen. In conclusion, ongoing research and development efforts in personalized cancer vaccines hold promise for enhancing treatment outcomes, and further exploration of optimization strategies and combination approaches will contribute to the advancement of cancer immunotherapy (Table 3).

### 3.4. Cytokine-Based Therapies

Cytokines serve as soluble components of the immune system, facilitating cell-to-cell communication and regulating various aspects of the innate and adaptive immune response. While elevated levels of certain cytokines like IL-6, IL-8, and TGFβ are associated with poor prognosis, there exist key cytokines that restrain tumor proliferation through anti-proliferative effects or by promoting cytotoxic activities [299,335,414]. To date, two cytokines have received FDA approval: IL-2 for renal cell carcinoma (RCC) and metastatic melanoma and IFNα for hairy-cell leukemia follicular non-Hodgkin lymphoma and melanoma. However, they are not utilized as monotherapy agents due to low clinical response rates and associated toxicity at high dosages. Instead, they are often used in combination with ICIs or other forms of immunotherapy [415]. Efforts to enhance the efficacy of cytokine-based therapies focus on modulating their activity by optimizing receptor affinity, improving serum half-life, enhancing tumor targeting, and reducing toxicity. The high-affinity receptors are expressed on Tregs and trigger Treg stimulation, whereas low-affinity receptors are mostly on NK and cytotoxic T cells [416]. To address this, mutations are introduced to generate muteins or superkines with altered affinities for IL-2 receptors, resulting in improved therapeutic indices [417,418].

Despite these advances, systemic toxicity remains a challenge with cytokine administration. Immunocytokines or antibody–cytokine conjugates for directed delivery of cytokine payloads are being investigated in preclinical studies and trials [419]. Additionally, a wide range of cytokines, such as IL-18, IL-2, IL-15, and IL-12, are in various stages of preclinical or clinical development in combination with other cancer immunotherapies [420]. Currently, IL-10, known for its immunosuppressive activities, is being tested in combination with chemotherapy and nivolumab, showing promise in enhancing anti-tumor cytotoxicity by CD8 T cells [421,422]. Pegilodecakin (pegylated recombinant human IL-10) in combination with a PD-1 inhibitor has shown promising activity in renal cell carcinoma (NCT02009449) (Table 4) [423]. Ongoing trials with bempegaldesleukin (NKTR-214; pegylated recombinant IL2) plus anti-PD1 (NCT02983045) continue to demonstrate well-tolerated and anti-tumor efficacy in metastatic urothelial carcinoma [424]. Overall, while cytokine therapy has been widely approved for use in combination with other therapeutic agents, its pleiotropic nature entails a wide range of impacts. The dynamic cytokine landscape across different cancer types and the optimization of dosage and administration methods are essential considerations to address challenges associated with cytokine-based therapies.

### 3.5. Oncolytic Viruses (OVs)

These genetically modified viruses, known as oncolytic viruses (OVs), are engineered to infect and selectively destroy tumor cells while sparing non-neoplastic cells. Currently, four OVs and one non-OV have received approval [425]. The first FDA-approved OV, Talimogene laherparepvec (T-VEC), is an engineered oncolytic herpes simplex virus type 1 (HSV1) used as a therapy for non-resectable metastatic melanoma. T-VEC replicates selectively in tumor cells, leading to tumor cell lysis and release of tumor antigens. This triggers a series of events, initiated by DCs, which present the antigens and prime T cells for anti-tumor responses, a process known as immunogenic cell death (ICD). To enhance safety, viral genes encoding virulence factors are deleted, and modifications prevent viral antigen processing and presentation, allowing only tumor cells to be presented on MHC-I. T-VEC is also engineered to express GM-CSF to promote local DC maturation and antigen presentation [426]. OVs can serve as agents for in situ vaccines to elicit anti-tumor responses. A Phase II trial conducted in resectable stage IIIB–IVM1a melanomas demonstrated efficacy, as evidenced by increased CD8 infiltration in tumors, establishing its potential role as a neoadjuvant [427]. Combining CAR-T therapy with IL-7-encoded or TGFbR II antibodies to promote oncolytic viruses in preclinical studies showed rapid tumor activity and cancer cell death. A study combining OVs with HER2-targeting CAR-T therapy is currently underway (NCT03740256). Additionally, T-VEC is being evaluated in combination with radiotherapy in sarcoma (NCT02453191) and melanoma as well as other solid tumors (NCT02819843).

### 3.6. Combined Approaches to Treat Hot, Altered, and Cold Immune Landscapes

As previously discussed, tumors employ intrinsic mechanisms involving signaling pathways such as Wnt-β catenin, NF-κB, PI3K/MAPK, and Notch signaling, alongside adaptive modulation of the TME following T-cell infiltration. In melanoma, Wnt-β catenin signaling impedes T-cell infiltration and fosters resistance to CTLA-4/PD-1 blockade [428]. In KRAS-addicted tumors, modulation of MAPK/PI3K or JAK/STAT3 systems regulates a milieu of cytokines/chemokines and their receptors, such as IL-6, CXCL8, TGFβ, IL1β, or CXCL1, crucial for immunosuppression via Treg differentiation, MDSC recruitment, and M1 to M2 conversion [429,430]. For more effective therapeutic interventions, comprehensive targeting of all components involved in immunosuppression and stratifying tumors based on their preexisting immunophenotype is imperative [431,432].

Hot tumors like melanoma or NSCLC, characterized by a preexisting tumor-infiltrating lymphocyte (TIL) population, are also associated with high tumor mutational burden (TMB) and immune checkpoints due to the ongoing anti-tumor response. Consequently, immune checkpoint inhibitor (ICI)-based approaches are particularly suitable. The non-redundant expression of PD-1 and CTLA-4 makes them promising targets for combination therapy, showing efficacy in advanced-stage melanoma, RCC, and NSCLC [284,433]. Introducing antagonists for other checkpoints like TIM3, TIGIT, LAG3, and CD39 in combination with PD-1/PD-L1 or CTLA-4 is yielding promising results. Several ongoing trials are investigating combination therapies using multiple immune checkpoints. Additionally, using PD-1 ICI as adjuvant therapy to prevent recurrence post-tumor resection has proven beneficial. In melanoma, pembrolizumab has demonstrated longer recurrence-free survival when used as adjuvant therapy [434]. Based on improved OS with the use of pembrolizumab as a neoadjuvant therapeutic agent, it has been approved for neoadjuvant therapy for resectable NSCLC. Alternatively, targeting costimulatory molecules like OX40 (also known as TNFRSF4 or CD134), TNFRSF7 (also known as CD27), CD28, TNFRSF9 (also known as 4-1BB ligand-receptor or CD137), and glucocorticoid-induced TNFR-related protein (GITR) with agonistic antibodies could be beneficial. This approach enhances T-cell expansion while simultaneously depleting regulatory T cells [435,436]. However, the results of CD28 superagonist with life-threatening cytokine release syndrome (CRS) underscore the challenge of selecting the right costimulatory molecule and dosage [436,437].

In immune-altered tumors, numerous immunosuppressive factors exert a significant influence. Dysregulated chemokine signaling alters the chemotactic recruitment of both anti-tumor and protumor cells, including the suppression of T cell-recruiting chemokines/receptors like CXCL9, CXCL10, CXCL11, CXCL13, CX3CL1, and CCL2, which can be epigenetically regulated or involve pathways that increase CXCL8-CXCR1/2 signaling, recruiting MDSCs and neutrophils to the TME [438]. Combination therapy with adagrasib and pembrolizumab has demonstrated increased CD8 infiltration and anti-tumor response by downregulating CXCL1, CXCL8, VEGF, and CD73, reshaping the TME and facilitating TIL infiltration, [439]. Pro-angiogenic cytokines like VEGF can suppress DC maturation, regulate TCR signaling, and inhibit IFN-γ and GNLY secretion, impacting immune responses beyond angiogenesis. VEGF/PD-1 bsAb in combination with chemotherapy has shown anti-tumor response in advanced NSCLC and EGFR-TKI-failed NSCLC, with ongoing Phase III trials (NCT05184712) [440]. Additionally, the combination of uliledlimab (anti-CD73) and toripalimab (anti-PD-1) has demonstrated increased ORR in NSCLC cohorts, with ongoing trials [NCT04322006] (Table 1). Other components of metabolic pathways maintaining an immunosuppressive environment, such as IDO and cytokines like IL-10 and TGFβ, impair anti-tumor immune responses by disrupting DC differentiation, migration, and antigen presentation, necessary for T-cell priming, making them promising targets for anti-tumor responses [441,442]. CD40 on APCs interacting with its ligands on helper T cells can induce APC activation and increased response from T cells. CD40 agonists are performing well in preclinical cancer models, leading to ongoing clinical trials evaluating CD40 agonists alone and in combination with immune checkpoint inhibitors (NCT04495257) (Table 1) [443,444].

In cold tumors, a proposed strategy involves combining various agents to prime T cells, induce anti-tumor T-cell infiltration, and remove co-inhibitory factors to counteract the immune-suppressive environment, thereby converting the cold tumor into a hot one [445]. This multifaceted approach may include therapeutic interventions such as vaccines, adoptive T-cell transfer (ACT) with the elimination of co-inhibitory signals, or the provision of co-stimulatory signals (e.g., anti-OX40 or anti-GITR). Genotoxic chemotherapies can induce neoepitopes by promoting mutations in cells and enhancing adjuvanticity through the generation of danger-associated molecular patterns (DAMPs) due to increased cell death [256]. Radiotherapy can also stimulate immune cells by inducing tumor cell death and generating DAMPs. In this regard, the inherent detection mechanisms of tumors and innate immune cells, notably involving cGAS/STING—a cytosolic DNA sensor—have demonstrated utility in increasing anti-tumor response. The accumulation of DNA resultant from radiotherapy or chemotherapy can activate the cGAS/STING pathway in DCs and tumor-associated macrophages (TAMs), modulating IFN-gamma levels in DCs and thereby facilitating CD8 infiltration [446]. Presently, STING agonists are undergoing evaluation in conjunction with immune checkpoint blockade (ICB) to exploit STING signaling for activating anti-tumor responses, with some demonstrating efficacy and a manageable safety profile (NCT03010176) [447]. Nonetheless, several of these agonists were halted in later development stages. Presently, novel STING agonists are under scrutiny, typically combined with dual blockade of PD-1/PD-L1 and CTLA-4 or one of them (NCT03956680, NCT03843359; NCT04096638).

Neoadjuvant chemotherapy has been shown to increase the CD8 T cell-to-Foxp3^+^ Treg ratio in TNBC [448]. Targeted therapies against tumor cells, such as HER2 and Trop-2 for TNBC and Nectin-4 for bladder cancer, can be utilized for targeted tumor cell killing. Enhanced cell death can contribute to increased DAMPs and inflammation. Treatment with a combination of tucatinib and ADC ado-trastuzumab emtansine has shown improved progression-free survival (PFS) in HER2-negative breast cancer as a primary endpoint in a Phase III trial (NCT03975647) [449]. However, one of the challenges in cold tumors will be the recruitment of the engineered cells in the tumor. A recent study in a murine melanoma model demonstrated a failure in the trafficking of CAR-T cells in the tumor due to the absence of T-cell trafficking signals CXCL9 and CXCL10, produced by DCs [450]. Hence, before heating up the tumor, ACT might not be the best approach for cold tumors. Another potential approach to “heat up” a cold tumor is targeted ICD of the tumor cells by using oncolytic viruses. The combination of oncolytic viruses with ICIs has demonstrated efficacy in unresectable melanoma compared to ICIs alone [451]. This was accompanied by increased CD8^+^ T-cell infiltration [452,453]. An oncolytic virus-based therapeutic agent that uses an inactivated herpetic virus to directly deliver a gene to cancer cells, followed by an anti-herpetic therapy with valacyclovir (Valtrex), killing the cells containing the gene. This can act both as an oncolytic virus and a therapeutic vaccine and is currently being investigated in a Phase III trial in prostate cancer (NCT01436968) (Table 4). With the development of therapeutic personalized cancer vaccines (PCVs) alongside preventive vaccines, the expansion of neoepitope-specific CD8 T cells emerges as a promising therapeutic avenue. However, the tolerizing effect of neoantigens on the T-cell population requires further elucidation. A recent study revealed a high frequency of predicted mutated neoantigens in HLA-A1 hotspots within the CD8 T cell-excluded zones of tumors, prompting inquiry into the possible tolerogenic effect of neoantigens on dampening immune responses [454].

A recent report has highlighted a significant decrease in the overall utilization of ICIs targeting PD-1/PD-L1 from Phase I to III trials. However, there is a notable shift in Phase I trials towards evaluating diverse treatment modalities like engineered cell therapies and bispecific antibodies, alongside exploring combinatorial approaches [455] (Table 5). Intriguingly, the decline in new trials employing PD-1/PD-L1 inhibitors has been observed since 2022 in skin cancer, respiratory thoracic cancer, and head and neck cancer, while there has been an increase in breast cancer and digestive tract-associated cancers. Many of these combination therapies and bispecific antibody-based approaches still involve targeting the PD-1/PD-L1 pathway, underscoring its continued dominance in the field [456].

**Figure 2:**
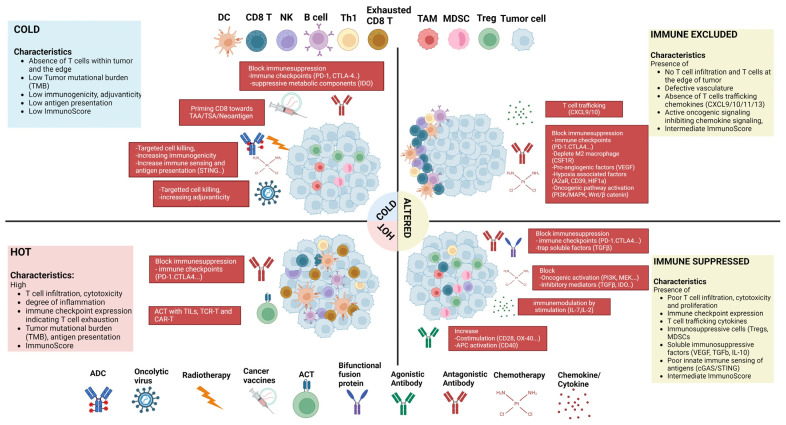
**Transforming cold tumors into hot—immunophenotypes and therapeutic strategies:** Tumors are categorized into four main types based on CD3 and CD8 populations: immune cold/desert, immune altered (excluded and suppressed), and immune hot/inflamed. The illustration outlines the major immune cell types regulating each tumor landscape and proposed therapeutic approaches, including existing and investigational interventions targeting the challenges within each immunophenotype. Cell types such as TAM, MDSC, Treg, and tumor cells primarily regulate immune suppression, while DC, CD8 T, NK, B, and TH1 cells mediate anti-tumor responses. Therapeutic approaches are depicted at the bottom, and each phenotype is described in four boxes along the edges. Each therapeutic agent is utilized either alone or in combination with others to effectively tackle the challenges presented by each phenotype. Abbreviations: DC: Dendritic cell; CD8 T: Cytotoxic T cell; NK: Natural killer cell; Th1: Type 1 helper cell; TAM: Tumor-associated macrophage; MDSC: Myeloid-derived suppressor cell; Treg: Regulatory T cell; ADC: Antibody–drug conjugate; ACT: Adoptive cell transfer therapy (created with BioRender.com; accessed on 1 March 2024).

Comprehensive targeting of all components involved in immunosuppression, alongside stratifying tumors based on their preexisting immunophenotype, is crucial for effective therapeutic interventions (Figure 2).

## 4. Overcoming Cancer Immunotherapeutic Resistance

Shifting the focus from directly attacking cancer cells to triggering the body’s innate anti-tumor response has shown promising results in clinical trials, albeit accompanied by a distinct set of challenges. Scientific advancements have spurred the development of various immunotherapeutic approaches. Among these, CPI and ACT have demonstrated significant efficacy. Furthermore, early indications suggest the potential effectiveness of therapeutic cancer vaccines [457]. In cases of hematological malignancies, CAR T-cell therapy has exhibited promising outcomes. Understanding tumors necessitates a thorough exploration of their inherent complexity and heterogeneity. Resistance to treatment encompasses a myriad of factors, including intricate metabolic, inflammatory, and neovascular mechanisms. Despite best efforts, many of these mechanisms remain elusive, warranting further investigation. By meticulously analyzing resistance patterns in patients and gaining a profound understanding of how tumor cells adapt, researchers have successfully categorized different forms of immunotherapy resistance. It is crucial to recognize that the response to therapy is a dynamic process that evolves with the progression of the patient’s disease [458]. Several factors can influence this, including physiological changes and the efficacy of treatment methods targeting the tumor(s). Some patients may not respond to immunotherapy, a condition termed “primary immune resistance,” which occurs when tumor antigens are absent, preventing T cells from recognizing them. It is conceivable that tumors may have developed mechanisms to conceal antigens within their microenvironment, thereby evading detection and response by the adaptive immune system. Such resistance hampers the body’s ability to combat tumors. Unfortunately, some patients who initially respond favorably to immunotherapy may later experience relapse, marked by the emergence of new metastatic sites. This phenomenon is termed “acquired or secondary immune resistance” [459]. Resistance can be further classified as intrinsic or extrinsic to cancer cells. Intrinsic resistance occurs within the tumor cell itself and involves various characteristics such as gene expression, cell signaling, immune recognition, and DNA damage response. Extrinsic resistance is observed in the surrounding microenvironment or in the systemic circulation during the T-cell bioactivation process.

### 4.1. Alterations in the Tumor Microenvironment (TME)

The TME showcases remarkable complexity and diversity, particularly in the advanced stages of solid tumors. It is imperative to highlight that genetic patterns within cancerous cells significantly contribute to the observed variations in cancer proximity [4,460]. The dynamic interplay among TME alterations and multiple genetic alterations in signaling pathways, as seen in dysregulated KRAS signaling pathways, shapes a complex landscape that profoundly influences tumor progression and immune evasion [430,461]. Understanding these intricate interactions is also crucial for devising effective therapeutic strategies aimed at disrupting this intricate balance and enhancing anti-tumor immune responses. Furthermore, numerous studies have shown that the rapid dissemination of cancer cells initiates a cascade of irreversible events. One such event is hypoxia, which triggers a complete metabolic transformation in cancer cells and prompts the TME to adapt to these new conditions. Through investigations into cellular interactions and vascular system development, researchers have illustrated how tumor cells can manipulate their surrounding environment’s components, facilitating the dissemination of cancer cells to distant sites [462]. During tumor formation, alterations in the surrounding environment influence both the growth of cancer cells and TME components, thereby impacting overall tumor progression [4,463,464]. The TME plays a pivotal role in directing various highly effective therapeutic strategies, including immunotherapies, antiangiogenic medications, treatments targeting cancer-associated fibroblasts (CAFs) and the ECM, as well as innovative modalities such as cold atmospheric plasma, oncolytic viral therapy, bacterial therapy, nanovaccines, and combination therapies involving repurposed pharmaceuticals. Interactions among cells within the TME have undergone extensive investigation and have been identified to play a pivotal role in immune response suppression [5]. Signaling between tumor cells and CD8^+^ T cells via the PD-L1/PD-1 pathway has been observed across various cancer types. However, besides tumor cells, several other cell types significantly hinder T-cell cytotoxicity, including Tregs, MDSCs, and TAMs. These cell subsets exhibit aberrant differentiation and heightened expression of immunosuppressive-associated proteins, fostering an anti-inflammatory environment that leads to CD8^+^ T-cell dysfunction and immune resistance. Tregs, a specific subset of CD4 T cells, are present in the thymus or peripheral blood [465], and they facilitate tumor growth within the TME by suppressing the immune response against colorectal cancer (CRC) through direct cellular interactions or cytokine and metabolite release. Research suggests that Tregs regulate the immune response through diverse mechanisms [465,466]. The TME contributes significantly to immune evasion mechanisms, influencing the activity and expression of the immune checkpoint network, particularly in aggressive diffuse large B-cell lymphoma (DLBCL) and indolent chronic lymphocytic leukemia (CLL).

### 4.2. Spatial Immune Cell Heterogeneity

Tumor heterogeneity poses a significant obstacle to achieving clinical success in immuno-oncology for cancer treatment. The complex dynamic platform between cancer cells and immune cells profoundly influences tumor progression and treatment response [467,468]. The genetic and histological characteristics of tumors exhibit heterogeneity, characterized by variations in cellularity, angiogenesis, extravascular ECM, and areas of necrosis within the tumor. Tumors exhibiting significant intratumoral heterogeneity have been demonstrated to have a less favorable prognosis, perhaps due to inherent aggressive characteristics or resistance to treatment [469,470]. Spatial heterogeneity of immune cells refers to the diversity in composition, distribution, concentration, and functional status of immune cells within a tissue or tumor. This heterogeneity significantly influences the clinical response to immunotherapy in cancer. Local alterations such as regional stiffness, coexistence of vascularized and hypoxic areas, spatial genotype differences, and differential immune cell distribution contribute to the complex interplay [471]. Cancer cells can evade both innate and adaptive immune responses, while ICIs have demonstrated potent anti-tumor immune responses. However, the spatial heterogeneity of the TME significantly impacts the distribution of tumor-infiltrating immune cells, resulting in variable degrees of immune evasion phenomena [472,473]. An analysis of the TME and identification of factors influencing its heterogeneity present a promising avenue for the development of immunotherapy biomarkers and the formulation of strategies to overcome acquired resistance to cancer therapeutics. In their study, Sobti et al. conducted intra-tumor spatial profiling to investigate the immune microenvironment in 42 biopsies from patients diagnosed with nasopharyngeal cancer (NPC). Their analysis unveiled spatial heterogeneity and identified immune-related biomarkers. Notably, protein targets associated with B cells (CD20), macrophages (CD68), Treg cells (PD-1, FOXP3), and NK cells (CD56) exhibited significant differential expression within CD45^+^ segments located in regions of immune-rich cancer cell islet compared to the adjacent stromal leukocytes. Conversely, markers linked to suppressive myeloid cell populations (VISTA, B7-H3, CD163) and T cells (Tim-3, LAG3, CD4) showed heightened expression levels within CD45^+^ segments of the surrounding stromal leukocytes compared to regions comprising immune-rich cancer cell islets [474]. Another study has identified four distinct immune phenotypes of ccRCC, unveiling their specific immune cell subtypes and cellular neighborhoods. These findings elucidate why some patients exhibit diverse survival outcomes owing to the spatial heterogeneity of the TME. Early intervention is necessary for macrophage/T-clustered patients. The identification of intercellular interaction functional units could help in prognosis evaluation and guide diagnosis and treatment [475]. Spatial immune heterogeneity is crucial to augment the efficacy of immunotherapies and surmount therapy resistance, thereby advancing more effective cancer treatment strategies [476].

### 4.3. Alterations in Antigen Presentation

Peptides generated from a cell’s expressed genes are captured by MHC I molecules, which subsequently present this antigenic information on the cell surface. This process facilitates the recognition of pathogenic cells by CD8 T cells. Many malignancies must develop defenses against CD8 T-cell destruction to initiate and propagate [477]. Understanding the mechanisms through which various cancers evade immune surveillance requires a fundamental comprehension of the MHC I pathway of antigen presentation. Peptides presented by MHC I are derived from the regular breakdown of cellular proteins along this pathway. The ubiquitin–proteasome system is responsible for continuously degrading endogenously produced proteins into oligopeptides [478]. This catabolic pathway facilitates the initial cleavages, particularly the appropriate C-terminal incision, necessary for generating the majority of peptides presented by MHC I [479,480]. A truncating mutation in the β2M gene, which is associated with MHC I and essential for its folding and transportation to the surface of T cells, has been linked to acquired resistance to anti-PD-1/PD-L1 therapy [268,481]. The immunohistochemical study of MHC-I heavy chains in the patient’s tumor samples revealed a loss of adventitial localization in the tumor compared to both the tumor and nearby stroma before treatment, despite continued production of MHC-I molecules in the recurrent tumor [482]. Recently, it has been showed that MHC class I loss is not primarily responsible for resistance to CPI treatment. This assertion is supported by the common occurrence of β2M and HLA-A loss in malignancies, which are typically reversed post-immunotherapy. The study emphasizes the dynamic nature of HLA-A and β2M expression in tumors, influenced by factors such as indication, metastatic status, immunophenotype, and immunotherapy treatments [483].

According to studies, rectifying deficiencies in antigen processing and presentation can significantly enhance the effectiveness of immunosuppressive therapy (ICI). In models of ICI resistance, whether primary or acquired, antagonizing cIAP1/2 has been shown to impede tumor growth [484]. The activation of noncanonical NF-κB signaling triggers immunological responses dependent on T cells, even in tumors devoid of β2M. This suggests that the direct recognition of tumor cell-expressed MHC class I by CD8 T cells is unnecessary. Conversely, lymphotoxin derived from T cells alters the genetic profile of both murine and human macrophages, making them capable of tumor destruction. In wild-type mice, cIAP1/2 antagonism reduces tumor burden by enhancing the phagocytosis of live tumor cells, but this effect is absent in animals lacking antigen-specific T-cell responses. The synergy achieved through CD47 blockage enhances efficacy, leading to the activation of a noncanonical NF-κB pathway that triggers a T cell–macrophage axis. This axis effectively inhibits the growth of malignancies resistant to checkpoint blockage, particularly in cases where MHC class I or IFN-γ sensing is absent. These findings present a potential strategy for managing checkpoint-resistant malignancies [484]. Trametinib, a small molecule MEK inhibitor, demonstrated notable effects in a mouse model of MAPK-activated HNSCC. It reduced extracellular signal-regulated kinase phosphorylation, increased MHC-I and PD-L1 expression and successfully overcame monotherapy resistance [485]. Chloroquine was found to enhance the expression of MHC-I antigens, rendering tumors more susceptible to dual immune checkpoint suppression through the administration of anti-CTLA-4 and anti-PD-1 antibodies [269]. Yamamoto et al. demonstrated that enhanced autophagy or lysosome function contributes to immune evasion by specifically targeting MHC-I molecules for degradation. This finding supports the rationale for employing a combined strategy of autophagy suppression alongside dual ICB therapy in the treatment of PDAC [269]. The ASPIRE nanovaccine, derived from DCs infected with recombinant adenovirus, holds promise in enhancing antigen delivery to lymphoid organs while also eliciting broad-spectrum T-cell responses for the elimination of existing malignancies [486]. A novel immunotherapeutic strategy, DT-Exo-STING, employs DC-derived chimeric exosomes loaded with STING agonists to enhance tumor-specific T-cell immunity. This innovative approach effectively reverses immunosuppressive glioblastoma microenvironments, inducing pro-inflammatory, tumoricidal conditions. Furthermore, it enhances sensitivity to ICB therapy and establishes systemic immune memory against post-operative glioma recurrence, marking a promising avenue for glioblastoma immunotherapy [487]. Additionally, treatments targeting TLRs have shown efficacy in promoting DC maturation and mitigating resistance to anti-PD1/PDL1 therapies.

### 4.4. Altered Signaling Pathways

The prognosis and sensitivity of cancer patients to immunotherapy can be significantly influenced by gene mutations and abnormalities in signaling pathways. Immunotherapy heavily relies on the INF-γ signaling cascade, which also plays a crucial role in resistance to ICI treatment [268,488,489]. A recent study showed that the master transcription factor TP63 in squamous cell carcinomas (SCCs) suppresses IFN-γ signaling, resulting in increased infiltration of CD8^+^ T cells and enhanced tumor killing. Silencing TP63 has been shown to enhance the anti-tumor efficacy of PD-1 blockade by promoting the infiltration and functionality of CD8^+^ T cells. Overexpression of TP63 might serve as a biomarker for SCC patients undergoing ICB therapy, and targeting the TP63/STAT/IFN-γ axis could potentially enhance the efficacy of ICB therapy [490].

In the cancer microenvironment, NK cells, T cells, and activated T cells secrete IFN-γ, a crucial cytokine that regulates the immune response via downstream enzymes JAK1/2 and signal transducer and activators of transcription (STATs) [491]. Mutations in JAK1/JAK2 within the downstream signaling pathway of IFN-γ are increasingly recognized as significant contributors to acquired resistance to ICI treatment. The heterodimeric IFNGR1/IFNGR2 receptor complex on tumor cells binds to IFN-γ released by tumor-specific T lymphocytes, activating JAK1 and JAK2, which subsequently phosphorylate a transcription factor known as STAT1. Phosphorylated STAT1 homodimers then translocate to the nucleus, modulating the transcription of IFN-γ-stimulated genes. This process enhances the synthesis of chemokines, attracting immune cells and directly inducing apoptosis in tumor cells [492,493]. Anti-tumor immune responses are influenced by the INF-γ axis in both positive and negative manners [494]. Firstly, it enhances antigen presentation through increased MHC-I secretion. Secondly, it recruits additional immune cells by upregulating the expression of chemokines (CXCL9, CXCL10, and CXCL11), which exert a potent chemoattractant effect on T cells [495]. Thirdly, it directly inhibits the proliferation and promotes apoptosis of cancer cells [492]. Conversely, IFN-γ operates via a negative feedback loop to increase the expression of PD-L1 and other essential immune-suppressive factors, such as IDO1, thereby diminishing the cytotoxic response and fostering adaptive resistance in cancer cells [496,497]. Moreover, IFN-γ/JAK signaling can upregulate the expression of PD-L1, enhancing antigen presentation and responsiveness to PD-1 antibodies. Additionally, it stimulates proteasome subunits and transporters associated with MHC-I [498,499].

After a median objective response duration of 1.8 years to pembrolizumab, disease progression occurred in two out of four melanoma patients. Whole-exome sequencing of their tumor tissues obtained at baseline and after disease progression revealed over 90% truncating dysfunctional mutations in both JAK1 (Q503* nonsense mutation) and JAK2 (F547 splice-site mutation). This was concurrent with the deletion of the wild-type allele and duplication of the mutant allele. The deactivation of JAK1 and JAK2 signaling resulted in the development of IFN-γ resistance, compromising immune surveillance and promoting tumor cell growth [268]. Inhibitors targeting the JAK-STAT pathway have shown potential in the treatment of lung cancer, facilitating the development of precise therapeutic strategies. Jia et al. presented a comprehensive overview of the JAK-STAT pathway, covering its composition, activation mechanisms, dynamic interactions, and the molecular drivers behind its abnormal activation in lung cancer (LC). They also highlighted recent advances in targeting this pathway for LC treatment, underscoring the necessity for further exploration into this promising therapeutic avenue [500]. Multiple investigations have demonstrated that the inability to upregulate PD-L1 and MHC-I expression leads to resistance to CTLA-4 and PD-1 inhibitors in instances of lost JAK/STAT signaling [493,498]. It is concerning that high-frequency loss-of-function mutations in malignancies could be associated with primary resistance to ICI therapy; as many as 19% of pretreatment melanoma biopsies exhibited mutations in the IFN-γ pathway [493,501]. Furthermore, exposure to IFN-γ can lead to mutations in this pathway, potentially resulting in the absence of PD-L1 expression, thus conferring resistance to PD-1/PD-L inhibitors [493]. The potential impact of mutations and deletions in STAT1 and other proteins associated with the IFN-γ pathway, such as IFNGR1 and IFNGR2, on resistance to ICIs needs further investigation [501]. A study demonstrated that an IFN signaling-deficient model of B16 murine melanoma showed efficacy against tumors with JAK2 loss but not those with JAK1 loss. However, overexpression of NLRC5, a receptor family caspase recruitment domain-containing nucleotide-binding oligomerization domain, restored the efficacy of adoptive cell therapy against B16-JAK1 loss tumors [502].

In addition to these signaling pathways, dysregulated Toll-like receptor (TLR) signaling pathways in cervical cancer stimulate the production of inflammatory cytokines and chemokines, thereby promoting tumor growth and metastasis. Consequently, aberrant TLR signaling cascades contribute to tumor evasion from immune surveillance [503]. Furthermore, mutations in signaling pathways such as K-RAS, which are also prevalent across various cancer types, have been associated with significantly increased expression of ICMs [430]. These dysregulated signaling pathways and mutations promote immune evasion, making tumors harboring them promising candidates for immune checkpoint blockade.

## 5. Discussion and Future Perspectives

Understanding the occurrence and progression of cancer necessitates an exploration of the intricate processes and immune evasion mechanisms that impede the body’s natural defenses. Immunotherapy, an innovative approach to cancer treatment, has transformed the therapeutic landscape by effectively leveraging the immune system to combat cancer, thereby significantly enhancing patient outcomes. The FDA has approved numerous cell therapy products, including those targeting CD19 and BCMA, owing to the efficacy of CAR-T cell therapy [359,458]. Scientific advancements in autologous treatments, such as CAR-T cell therapy, have showcased their effectiveness. However, the limitations posed by HLA restrictions impede their widespread application, contributing to the substantial costs and time commitments associated with manufacturing cellular therapies. Further exploration is warranted for CAR-NK cells, a more innovative therapeutic approach [378]. Antibodies targeting PD-1, PD-L1, and CTLA-4 have demonstrated promising therapeutic outcomes across various tumor types and patient populations. Nevertheless, solid tumors encounter obstacles that impede their efficacy, including an immunosuppressive TME, inadequate trafficking, and a diverse array of tumor antigens [151,152]. Understanding the intricate impact of high intra-tumoral pressure on treatment delivery presents a multifaceted challenge. Recent breakthroughs in cancer immunotherapies, encompassing ICIs and CAR-T, have transformed our approach to treating both solid and hematologic malignancies. However, these therapies exhibit distinct toxicity profiles owing to their mechanisms of action [320]. To achieve improved outcomes with these therapies, additional preclinical research is imperative to augment the efficacy and responsiveness of immune checkpoint inhibition while mitigating potential adverse effects. With the help of scientific research, researchers have achieved notable progress in the realm of immune checkpoint therapy.

Harnessing antigens to stimulate the human immune system against malignant tumors, cancer vaccines have emerged as a significant advancement. Various types of cancer vaccines have emerged, including oncolytic viruses (OVs), cellular vaccines, peptide vaccines, and nucleic acid vaccines [504]. OVs, specifically, are viruses capable of infecting and replicating within cancer cells, leading to the destruction of infected cells and the stimulation of a broader immune response against tumors. Recognized for their diverse anti-tumor mechanisms, OVs show promise as cancer treatment options [425]. They function by inducing cell destruction, activating the immune system, triggering inflammation, and modulating energy metabolism. Although OVs have exhibited encouraging outcomes in clinical trials across different cancer types, overcoming resistance to their therapeutic effects remains a challenge. Integrating OVs with conventional treatments like chemotherapy, immunotherapy, targeted therapies, and cellular therapies is crucial for augmenting response rates [505]. Cellular vaccines, including DC therapy and CAR-T therapy, play a crucial role in enhancing anti-tumor immunity by harnessing remarkable abilities for antigen recognition, processing, presentation, and T-cell sensitization [506]. Nevertheless, the majority of DC vaccines necessitate the isolation of DC cells or DC mononuclear progenitor cells from patients, a process demanding precision and consuming significant time. To mitigate these challenges, scientists have achieved notable progress in the development of nucleic acid vaccines, such as DNA and mRNA vaccines, aimed at circumventing the complexities associated with synthesizing macromolecules in laboratory settings [507].

Moreover, leveraging high-throughput sequencing alongside multi-omics methodologies is essential for elucidating the tumor microbiome and its relevance in cancer immunotherapy. Advanced NGS technologies provide intricate taxonomic and functional profiles of microbial communities. This facilitates the identification of hitherto undiscovered species and enables differentiation between various strains at a molecular level. Diverse omics approaches, such as metatranscriptomics, metaproteomics, and metabolomics, offer invaluable insights into microbial metabolism, host immune response, and cancer progression. The integration of machine learning algorithms and bioinformatics tools is pivotal for the analysis and interpretation of multi-omics data, thereby playing a pivotal role in formulating personalized cancer treatment strategies [508,509]. Single-cell visualization techniques employing multiplexed single-molecule RNA-FISH (smRNA-FISH) and immunofluorescence (IF) have garnered considerable attention in high-throughput imaging platforms. These methods facilitate a comprehensive examination of interactions between tumor and immune compartments at the single-cell level, offering valuable insights into phenotypes, tumor heterogeneity, and cellular interactions [510]. A deep understanding of the TME is essential for the successful execution of clinical trials centered on immunotherapy. Employing a rigorous scientific methodology and analyzing patient TME samples at baseline and at various time points can enhance the efficacy of clinical trials. Utilizing mathematical models, images generated through multiplexed IF can unveil the detectable activity of T cells expressing ICMs. This activity can then be correlated with single-cell RNA sequencing data, offering invaluable insights [511].

A recent review underscores the growing interest in harnessing mRNA for cancer treatment. Amidst the COVID-19 pandemic, the utilization of lipid nanoparticle technology in mRNA vaccines has yielded promising results. Recent advancements in mRNA and nanoformulation-based delivery technologies have brought attention to their potential in cancer immunotherapy [512]. Through rigorous scientific exploration, nanotechnology holds the potential to revolutionize cancer immunotherapy by addressing the challenges presented by tumor complexities, potential off-target side effects, and low immunogenicity [513]. The development of cellular nanocarriers capable of precisely delivering small immunological molecules to targeted areas will thereby unlock new avenues for precise drug delivery. However, their efficacy is hindered by the lack of precise control over their targeting capabilities. The practical application of nanomaterials in medicine faces obstacles such as limited drug delivery to tumors, potential toxicity concerns, and challenges related to drug metabolism in the body. Continuous exploration and refinement of state-of-the-art measurement and characterization techniques are essential to ensure the effective integration of nanomaterials into cancer immunotherapy [514,515]. Ultimately, immunotherapy presents significant advantages over conventional chemoradiotherapy and targeted therapy. However, it is crucial to delve into the complexities of immune resistance mechanisms to improve the survival outcomes for individuals combating cancer. With ongoing advancements in tumor immunology, bioinformatics, and sequencing technologies, scientists anticipate the emergence of even more promising immunotherapy strategies.

## Figures and Tables

**Table 1:** Selected ongoing trials in antibody-based therapies across different cancer types (clinicaltrials.gov, accessed on 1 March 2024).

Condition	Intervention	Format	Gov. Identifier	Phase	Status	Enrollment
**Immune checkpoint inhibitors**						
Metastatic hepatocellular carcinoma (HCC)	Cobolimab (anti Tim3), Dostarlimab (anti PD-1)	Humanized IgG4	NCT03680508	II	Recruiting	42
Advanced solid tumor	BND-22 (anti-LILRB1) monotherapy or in combination with Pembrolizumab (anti-PD-1) or Cetuximab (anti EGFR)	Humanized IgG4	NCT04717375	I/II	Recruiting	456
Stage IV NSCLC	Nivolumab Ipilimumab plus chemotherapy (Carboplatin Paclitaxel Pemetrexed Cisplatin)	--	NCT03215706	III	Active, not recruiting	719
Metastatic squamous HNSCC	Durvalumab in combination with small-molecule antagonists of CXCR2 and STAT3	IgG1	NCT02499328	I/II	Active, not recruiting	340
Hormone-sensitive prostate cancer	Nivolumab monotherapy or in combination with BMS-986253 (anti-IL8)	--	NCT03689699	I/II	Active, not recruiting	60
Advanced solid tumor	Uliledlimab (anti- CD73) Toripalimab (anti-PD-1)	Humanized mAb	NCT04322006	I/II	Recruiting	376
**Antibody** **–drug conjugates**						
HER2-positive and PD-L1-positive locally advanced or metastatic breast cancer	T-DM1 (Trastuzumab Emtansine) Atelizumab (anti PD-L 1)	-	NCT04740918	III	Active, not recruiting	96
Pre-treated HER2 breast cancer	T-Dxd (Trastuzumab Deruxtecan) Capecitabine Lapatinib Trastuzumab	-	NCT03523585	III	Active, not recruiting	608
Advanced solid tumor	SGN-B7H4V (B7-H4 targeted ADC)	-	NCT05194072	I	Recruiting	430
Relapsed or refractory DLBCL	Loncastuximab Tesirine in combination with Rituximab		NCT04384484	III	Recruiting	350
**Bispecific Antibodies (bsAb)**						
Advanced solid tumors	IOS-1002 (anti- LILRB1, LILRB2, KIR3DL1) monotherapy or in combination with Pembrolizumab	HLA-B57-Fc fusion protein	NCT05763004	Ia/Ib	Recruiting	140
	CDX585 (anti-LILRB2, PD-1)	IgG1k	NCT05788484	I	Recruiting	130
Advanced hepatobiliary cancer	Volrustomig (anti-PD-1, CTLA-4) Or Rilvegostomig (anti-PD-1, TIGIT) as monotherapy or in combination With Carboplatin Gemcitabine Cisplatin	IgG1 Humanized IgG1	NCT05775159	II	Recruiting	260
High risk locally advanced cervical cancer	Volrustomig (anti- PD-1, CTLA-4)	IgG1	NCT06079671	III	Recruiting	1000
EGFR-mutant locally advanced or metastatic non-squamous NSCLC	Ivonescimab (anti PD-1/VEGF)	Humanized IgG1	NCT05184712	III	Recruiting	470
Refractory small cell lung cancer (SCLC)	Tarlatamab (engages DLL3, CD3)	bsTCE	NCT05060016	II	Active, not recruiting	222
Metastatic castration-resistant prostate cancer (mCRPC)	Gammabody^®^ (engages Vγ2Vδ9, PSMA)	Bs γδTCE	NCT05369000	I/II	Recruiting	66
**Other monoclonal antibodies (mAbs)**						
Metastatic HCC	Humax-IL8 (anti IL-8) or Cabiralizumab (anti-CSF1-R) in combination with Nivolumab	Humanized IgG1K --	NCT04050462	II	Active, not recruiting	23
Treatment naïve advanced melanoma or RCC	Sotigalimab (CD40 agonist mAb)\ Nivolumab Ipilimumab	--	NCT04495257	I	Recruiting	36

**Table 2:** Selected ongoing trials in adoptive cell transfer (ACT) therapies across different cancer types (clinicaltrials.gov, accessed on 1 March 2024).

Condition	Intervention	Gov. Identifier	Phase	Status	Enrollment
**CAR-T**					
GD2-expressing brain tumor	GD2 CAR-T	NCT03373097	I	Recruiting	34
CLDN6-positive relapsed or refractory advanced solid tumors	CLDN6 CAR-T CLDN6 uRNA-LPX/CLDN6 modRNA-LPX (mRNA vaccine)	NCT04503278	I/IIa	Recruiting	145
Children with recurrent/refractory malignant brain tumors	IL13Ralpha2 targeting Hinge-optimized 41BB-co-stimulatory CD19-CAR-T Fludarabine Cyclophosphamide	NCT04510051	I	Recruiting	18
Multiple myeloma	SLAMF7 CAR-T BCMA CAR-T Bortezomib Dexamethasone Lenalidomide Cyclophosphamide Fludarabine	NCT04499339 NCT04923893	I/IIa III	Recruiting	38 650
CD30^+^ refractory/relapsed Hodgkin lymphoma (HL) and non-Hodgkin lymphoma (NHL)	CD30 CAR-T	NCT02690545	Ib/II	Recruiting	40
Relapsed or refractory B-cell lymphoma	CD19-CD22 CAR-T	NCT04715217	I/II	Recruiting	24
Malignant pleural disease	Mesothelin CAR-T Cyclophosphamide Pembrolizumab	NCT02414269	I/II	Active, not recruiting	113
CD70-expressing cancers	CD70 CAR-T	NCT02830724	I/II	Recruiting	124
**CAR-NK**					
Stage IV ovarian cancer, refractory testis cancer, recurrent endometrial cancer CAR NK	CLDN6 targeting CAR-NK cells	NCT05410717	I/IIa	Recruiting	200
Metastatic locally advanced gastric/GEJ cancer or HNSCC	PD-L1 t-haNK N-803 Pembrolizumab (anti-PD-1)	NCT04847466	II	Recruiting	55
Relapsed/refractory NHL, CLL) or B-cell acute lymphoblastic leukemia (B-ALL)	Allogenic CD19-CAR-NK	NCT05020678	I	Recruiting	150
Refractory metastatic colorectal cancer	NKG2D CAR-NK	NCT05213195	I	Recruiting	38
**CAR-Gamma Delta T cells**					
B-cell malignancy	CD20 Allogenic Gamma Delta CAR-T Fludarabine Cyclophosphamide	NCT04735471	I	Recruiting	78
**TCR-T**					
Induction therapy prior to definitive treatment (chemoradiation or surgery) of locoregionally advanced HPV-associated cancers	Conditioning, E7 TCR-T cells Aldesleukin (IL-2)	NCT05639972	I/II	Recruiting	15
TCR against neoantigens in subjects with relapsed/refractory solid tumors (gynecologic colorectal, pancreatic, NSCLC, ovarian cancer)	Neoantigen-specific TCR-T cell Aldesleukin	NCT05194735	I/II	Active, not recruiting	180
Previously untreated advanced melanoma	PRAM (PReferentially expressed antigen in Melanoma) TCR-T Nivolumab (anti PD-1) Nivolumab + Relatlimab (anti LAG3)	NCT05122221	III	Recruiting	12
Refractory mesothelin-expressing mesothelioma (MPM), ovarian cancer (OvC), cholangiocarcinoma (CHO), or NSCLC	Gavocabtagene autoleucel (Mesothelin targeted TCR-T) Fludarabine Cyclophosphamide Nivolumab Ipilimumab	NCT03907852	II	Recruiting	175
KRAS G12V-expressing solid tumors	KRAS G12V targeted TCR-T	NCT06105021	I/II	Recruiting	100
**Tumor-Infiltrating Lymphocyte (TIL) therapy**					
Metastatic CRC, ovarian, pancreatic, and breast cancer	Young TIL Pembrolizumab Aldesleukin Chemotherapy	NCT01174121	II	Recruiting	332
Recurrent, metastatic, or persistent cervical carcinoma	Autologous TIL (IL-145) Pembrolizumab IL-2	NCT03108495	II	Recruiting	189

**Table 3:** Selected ongoing trials in cancer vaccines across different cancer types (clinicaltrials.gov, accessed on 1 March 2024).

Condition	Intervention	Adjuvant	Covt. Identifier	Phase	Status	Enrollment
**mRNA vaccine**						
Advanced melanoma	Neoantigen mRNA Pembrolizumab	-	NCT05933577	III	Recruiting	1089
	TriMix DC (MAGE-A3, MAGE-C2, tyrosinase and gp100) Ipilimumab	CD70, CD40 ligand, TLR4	NCT01302496	II	Recruiting	39
	Neoantigen mRNA Pembrolizumab	--	NCT03815058	II	Recruiting	131
Resected Stage II (High Risk) and Stage III CRC	Neoantigen mRNA Pembrolizumab	-	NCT04486378	II	Recruiting	201
Unresectable recurrent or metastatic HPV16+ HNSCC	HPV 16 E6 and E7 mRNA Pembrolizumab	-	NCT04534205	II	Recruiting	285
NSCLC	NY-ESO-1, MAGEC1, MAGEC2, 5 T4, survivin, and MUC1 mRNA Durvalumab	-	NCT03164772	I/II	Recruiting	61
Advanced malignant solid tumors.	Neoantigen mRNA	-	NCT05198752	I	Recruiting	30
**DNA vaccine**						
Advanced hepatocellular carcinoma	Neoantigen DNA	Plasmid Encoded IL-12	NCT04251117	I/IIa	Recruiting	36
CRC	OncoMimics™ peptides, UCP2 Nivolumab	Montanide	NCT05350501	II	Recruiting	34
Resectable HPV Type 16- and/or 18-positive head and neck cancer	HPV16/18 E6/E7 DNA	Flt3L	NCT05286060	II	Recruiting	25
Early stage TNBC	MDM2, YB1, SOX2, CDC25B, CD105 plasmid	GM-CSF	NCT05455658	II	Recruiting	33
**Peptide vaccine**						
IIIC-IV melanoma or hormone receptor-positive Her2-negative metastatic refractory breast cancer	Neoantigen peptide Nivolumab	Poly ICLC	NCT05098210	I	Recruiting	20
Advanced solid tumor	Neoantigen peptide Pembrolizumab	--	NCT05269381	I	Recruiting	36

**Table 4:** Selected clinical trials with oncolytic viruses, and engineered cytokine-based therapies (clinicaltrials.gov accessed on 1 March 2024).

Condition	Intervention	Govt. Identifier	Phase	Status	Enrollment
**Oncolytic viruses**					
Pancreatic adenocarcinoma, ovarian, biliary, and colorectal cancer	LOAd703 (oAD/CD40L-4-1BBL)	NCT03225989	I/II	Active not, recruiting	46
Metastatic pancreatic cancer	VCN-01(oAD/HA) Nab-Paclitaxel Gemcitabine	NCT05673811	II	Active not, recruiting	96
Localized prostate cancer	ProstAtak (aglatimagene besadenovec + Valacyclovir	NCT01436968	III	Active, not recruiting	711
**Engineered cytokines**					
Metastatic castration sensitive and castration-resistant prostate cancer	NHS-IL12 (IL-12 molecules fused to anti-NHS76)	NCT04633252	I/II	Recruiting	86
Advanced Kaposi sarcoma	NHS-IL12 monotherapy or in combination with M7824 (anti PD-L1/TGFβ TRAP)	NCT04303117	I/II	Recruiting	64
Locally advanced or metastatic solid tumors	IL-15–IL-15Rα (sushi) heterodimer (IL15 superagonist)	NCT04250155	I	Recruiting	250
Advanced solid tumor	Pegilodecakin (PEG-rIL-10)	NCT02009449	I	Active, not recruiting	350

**Table 5:** FDA-approved combination immunotherapies.

Combination Agent	Conditions
Nivolumab + Ipilimumab	Metastatic melanoma, advanced RCC, MSI-H or dMMR metastatic CRC, advanced HCC, metastatic NSCLC (PD-L1 tumor expression ≥1%), unresectable malignant pleural mesothelioma, metastatic ESCC
Nivolumab + Chemotherapy	Metastatic ESCC, metastatic gastric cancer Neo
Nivolumab + Ipilimumab + 2 Cycles of Platinum-Doublet chemotherapy	First-line metastatic or recurrent NSCLC, esophageal or GEJ carcinoma
Nivolumab + Relatlimab-rmbw	Unresectable/metastatic melanoma
Pembrolizumab + Chemotherapy	HNSCC, metastatic SCLC, high-risk early-stage and locally recurrent unresectable/metastatic TNBC, advanced unresectable/metastatic HER2-negative GEJ adenocarcinoma, first-line metastatic non-squamous NSCLC, locally advanced unresectable/metastatic BTC
Pembrolizumab + Chemoradiotherapy	FIGO 2014 Stage III-IVA cervical cancer
Pembrolizumab + Axitinib	First-line advanced RCC
Pembrolizumab + Lenvatinib	Non-MSI-H or dMMR advanced endometrial carcinoma, first-line advanced RCC
Pembrolizumab + Chemotherapy + Bevacizumab	Metastatic cervical cancer
Pembrolizumab + Trastuzumab + Chemotherapy	First-line locally advanced unresectable/metastatic PD-L1-positive HER2-positive GEJ adenocarcinoma
toripalimab-tpzi + Chemotherapy	Metastatic or recurrent, locally advanced NPC
Durvalumab + Chemotherapy	Locally advanced or metastatic BTC
Durvalumab + Chemoradiotherapy	Unresectable Stage III NSCLC
Atezolizumab + Bevacizumab + Chemotherapy	First-line metastatic non-squamous NSCLC
Atezolizumab + Bevacizumab	First-line unresectable HCC
Atezolizumab + chemotherapy	First-line Extensive stage SCLC, metastatic NSCLC without EGFR/ALK aberrations Metastatic TNBC
Atezolizumab + Cobimetinib + Vemurafenib	BRAFV^600^ mutation-positive advanced melanoma
Avelumab + Axitinib	Advanced RCC
Avelumab + Chemotherapy	Locally advanced or metastatic UC
Tremelimumab + Durvalumab	Unresectable HCC
Tremelimumab + Durvalumab + Chemotherapy	Metastatic NSCLC
Daratumumab + bortezomib + dexamethasone + thalidomide	MM
Elotuzumab + Lenalidomide + Dexamethasone	Newly diagnosed MM
Efortumab vedotin-ejfv (ADC) + Pembrolizumab	Locally advanced or metastatic UC
Polatuzumab vedotin + Rituximab + Chemotherapy	previously untreated DLBCL
Anti-PD-1 (Nivolumab, Pembrolizumab, Toripalimab-tpzi), anti-CTLA-4 (Ipilimumab, Tremelimumab), anti-PD-L1 (Atezolizumab, Avelumab, Durvalumab), anti-LAG3 (Relatlimab), anti-VEGF (Bevacizumab), anti-HER2 (Trastuzumab), anti-CD38 (Daratumumab), anti-SLAMF7 (Elutuzumab), CD-79b directed ADC (Polatuzumab vedotin)	

Abbreviations: RCC: Renal cell carcinoma; CRC: Colorectal cancer; HCC: Hepatocellular carcinoma; NSCLC: Non-small-cell lung cancer; SCLC: Small-cell lung cancer; HNSCC: Head and neck squamous cell carcinoma; TNBC: Triple-negative breast cancer; ESCC: Esophageal squamous cell carcinoma; NPC: Nasopharyngeal carcinoma; GEJ: Gastroesophageal junction; BTC: Biliary tract cancer; UC: Urothelial carcinoma; MM: Multiple myeloma; DLBCL: Diffuse large B-cell lymphoma; MSI-H: microsatellite instability-high; dMMR: Mismatch repair deficient.

## Data Availability

The study did not report any new results or data.
